# Metabolic profiles in laryngeal cancer defined two distinct molecular subtypes with divergent prognoses

**DOI:** 10.3389/fimmu.2025.1512502

**Published:** 2025-05-22

**Authors:** Dan Zheng, Xuan Pu, XuHui Deng, Cui Liu, SiJun Li

**Affiliations:** ^1^ Department of Otolaryngology, Head and Neck Surgery, Affiliated Hospital of North Sichuan Medical College, Nanchong, China; ^2^ Department of Clinical Medicine, North Sichuan Medical College, Nanchong, China

**Keywords:** laryngeal cancer, molecular subtype, metabolic profiles, prognosis model, diagnostic model, ScRNA-seq

## Abstract

**Background:**

Laryngeal cancer (LCA) is the second most common type of head and neck malignancy, characterized by high recurrence rates and poor overall survival (OS). However, progress in curing LCA through molecular-targeted diagnostics and therapies is slow and limited. The occurrence and progression of cancer are closely associated with metabolic reprogramming. Therefore, this study aimed to identify metabolism-related LCA subtypes through a comprehensive analysis of transcriptomic, mutational, methylation, and single-cell RNA sequencing, in hopes of finding factors which influences the prognosis of LCA.

**Methods:**

First, to identify metabolism-related LCA subtypes, data from 114 patients with LCA from The Cancer Genome Atlas (TCGA) dataset were collected for an unsupervised clustering analysis, which focused on the expression characteristics of survival-related metabolic genes. Subsequently, prognostic and diagnostic models have been developed using machine learning techniques. Specifically, the prognostic model utilized the least absolute shrinkage and selection operator (LASSO) Cox regression, whereas the diagnostic model was built using the Random Forest (RF) algorithm. Furthermore, to ensure the reproducibility, the results of the subtypes and models were validated using three independent bulk RNA datasets and a scRNA-seq dataset.

**Results:**

Two robust subtypes were identified and independently validated. Each subtype has a distinct prognostic outcomes and molecular features. Specifically, the *LCA1* subtype exhibited better prognosis, enriched metabolic pathways, and higher mutation frequencies. Notably, significant damaging mutations in the methyltransferases *NSD1* were observed in this subtype. In contrast, the *LCA2* subtype was associated with poorer prognosis, higher immune infiltration, and elevated methylation levels. Moreover, in *LCA2* tumors, higher levels of T cell/APC co-inhibition and inhibitory checkpoints were observed. In addition, the diagnostic model demonstrated strong performance, achieving an area under the curve (AUC) values of 1.000 in the training group and 0.947 in the validation group. The prognostic model effectively predicted patient outcomes, with the RiskScore emerging as an independent prognostic factor.

**Conclusion:**

This study offers new perspectives for patient stratification and presents opportunities for therapeutic development in LCA. Furthermore, we explored the potentials of several key tumor markers for both diagnosis and prognosis prediction.

## 1 Introduction

As the second most common head and neck malignancy, Laryngeal cancer (LCA) accounted for over 184,615 new cases and approximately 99,840 deaths globally in 2020 (1). Although medical diagnostic and treatment methods are continuously evolving, the diagnosis of LCA still relies primarily on endoscopic and pathological examinations (2). LCA treatment typically involves surgery combined with radiotherapy and chemotherapy (3). Cetuximab, Pembrolizumab, and Nivolumab have been used in patients with LCA, particularly in cases of recurrence and metastasis (4–6). Endoscopic techniques such as transoral laser microsurgery (TLM) and transoral robotic surgery (TORS) are increasingly being employed for the effective removal of suitable early-stage LCA (7). However, owing to the concealed nature of the laryngeal site and the lack of early diagnostic methods, LCA is always diagnosed at advanced stages (III-IV) (8). This late diagnosis limits the patients’ therapeutic options and significantly affects their quality of life. Numerous studies have increasingly focused on exploring the molecular biomarkers of LCA, however, despite the identification of some potential molecular targets such as *TLSs*, *NOTCH1*, and *BMP2*, new effective therapeutic targets for LCA have not yet been confirmed (9–11). Hence, it is crucial to identify early diagnostic markers and explore the molecular mechanisms which affect the prognosis of LCA.

The metabolic processes of malignant tumors were different from those of normal tissues, because they require higher and faster amounts of materials to support the proliferation of tumor cells. Consequently, tumor cells tend to undergo metabolic reprogramming to meet these elevated demands (12, 13). Because of such differences between malignant tumors and normal tissues, it is possible to explore targeted therapies against the metabolic dependence of tumor cells. According to some scholars, several anti-tumor pathways targeting metabolic enzymes have been identified, including: 5-fluorouracil, Capecitabine, Pemetrexed, and Raltitrexed targeting to Thymidylate synthase (TS); Methotrexate, Pemetrexed targeting to Dihydrofolate reductase (DHFR); Pemetrexed targeting to Glycinamide ribonucleotide formyltransferase (GARFT), et al. (14). In addition, many studies have employed multi-omics strategies for metabolic subtype classification. Yuan et al. revealed the metabolic heterogeneity associated with *HER2* in gastric cancer in response to immunotherapy and neoadjuvant chemotherapy (15). Similarly, Li et al. employed a multi-omics analysis to reveal *TAM2*-related glycolysis and pyruvate metabolism remodeling in pancreatic cancer (16). Hepatocellular carcinoma was classified into three metabolic subclasses: C1, C2, and C3 (17). Similarly, uterine corpus endometrial carcinoma is classified into two types, C1 and C2 (18). However, the metabolism-related molecular characteristics of LCA remained unreported.

In this study, we utilized unsupervised clustering analysis to categorize LCA into two distinct subtypes based on metabolism-related gene expression profiles for the first time. The reproducibility of the two subtypes was confirmed using two independent datasets. Moreover, each subtype features by different somatic alterations, immune infiltration profiles, DNA methylation patterns, metabolic features, and clinical outcomes. We further developed diagnostic and prognostic models by employing the Random Forest (RF) and least absolute shrinkage and selection operator (LASSO) Cox regression methods.

## 2 Materials and methods

### 2.1 Data source

The data used in this study was primarily obtained from two public databases. RNA-seq data, clinical information, DNA methylation data, and DNA mutation data from 114 LCA samples and 12 matched normal mucosal samples were downloaded from The Cancer Genome Atlas (TCGA, available at http://cancergenome.nih.gov). This dataset was employed to identify of LCA subtypes, and the construction of diagnostic and prognostic models. Additionally, a single-cell RNA sequencing dataset (GSE252490) (19) and three bulk RNA sequencing datasets (GSE130605 (20), GSE27020 (21), GSE142083 (22) were acquired from National Center for Biotechnology Information’s Gene Expression Omnibus (GEO, accessible at https://www.ncbi.nlm.nih.gov/geo). These datasets were used to validate the reliability and generalizability of the findings. Specifically, GSE130605, which included 50 LCA tissue samples and 50 matched normal tissue samples, was used to validate the metabolic subtype results. GSE27020, containing 109 LCA samples, was used for both metabolic subtype validation and prognostic risk model validation. GSE142083, comprising 53 LCA samples and 53 matched normal samples, was employed to validate the diagnostic prediction models. GSE252490 consists of three LCA samples with lymph node metastasis and was used to validate metabolic subtypes, diagnostic prediction models, and prognostic risk assessment models at the single-cell level.

### 2.2 Discovery and confirmation of subtypes related to metabolism

The previous 2752 genes associated with metabolism were collected for further analysis (23). First, by selecting genes with high median absolute deviation values (MAD > 0.5) in the expression profiles of the TCGA dataset, we obtained 1534 genes. Using COX regression analysis of survival in the TCGA cohort with the R package “survival”, genes significantly associated with prognosis were identified (P < 0.05). These genes across all patients were then utilized for consensus clustering analysis with the R package “ConsensusClusterPlus” (24, 25). To identify robust clusters, the cumulative distribution function (CDF) and consensus heatmap were applied to choose the optimal K. Consequently, the TCGA dataset samples were divided into two subtypes, designated as *LCA1* and *LCA2*. Next, by using the R package “DESeq2” (26), differential gene expression analysis was conducted between the two subtypes. Genes with an absolute |log2 fold change (FC)| > 0.5 and an adjusted P-value < 0.05 were considered potential candidate genes. Finally, the same consensus clustering analysis was performed on datasets GSE130605 and GSE27020 using the same candidate genes to validate metabolism-associated subtypes in other cohorts.

### 2.3 Immunoinfiltration analysis

Single-sample GSEA (ssGSEA) was applied to estimate the relative fraction of 13 immune-related functions and 23 immune cells between the two subtypes by using the “Gene Set Variation Analysis (GSVA)” package (27). We assessed the infiltration of immune components, stromal components, and tumor components using the ESTIMATE algorithm, which provided the ImmuneScore, StromalScore, ESTIMATEScore, and TumorPurity (28). Additionally, we compared the expression levels of several immune checkpoint genes between the two subtypes (29).

### 2.4 Calculation of gene signatures related to metabolism

A total of 114 metabolism-relevant pathways were gathered from previously published research (17, 27). The R package “GSVA” (27) was used to quantify the enrichment degree of metabolism-relevant signatures for each sample. Gene Ontology (GO) and Kyoto Encyclopedia of Genes and Genomes (KEGG) (30) enrichment analyses were applied to the highly expressed metabolism-relevant genes of the two subtypes, respectively.

### 2.5 Characterization of mutations

Mutation annotation format (MAF) files for LCA were downloaded from the TCGA and processed by the “maftools” R package. Besides, mutation information was also analyzed by using the “maftools” R package (31) between the two subtypes.

### 2.6 Differences in DNA methylation between the subtypes

To explore the epigenetic differences between the two subtypes, methylation data for LCA was downloaded from the TCGA database. Differentially methylated CpG sites were identified by using the “limma” R package (32). Then, 1471 CpG probes were considered as the most differentially methylated sites between the two subtypes (Absolute |log2 fold change (FC)| > 0.25 and Adjusted P-value < 0.01). In order to better explore the epigenetic differences, GO enrichment analysis (30) was applied to the highly methylated genes of the two subtypes, respectively.

### 2.7 Identification of metabolism-associated diagnosis model for LCA

To explore a robust diagnostic model, we initially identified differentially expressed genes between tumor and normal samples from the TCGA dataset by using the R package “DESeq2” (26). By applying the stringent criteria of absolute |log2 fold change (FC)| > 2 and an adjusted P-value < 0.01, we successfully identified 293 differentially expressed genes. Subsequently, using the R package “randomForest” (33), we developed a diagnostic model to distinguish tumor tissues from normal tissues. A Nomogram (34) was generated to facilitate clinical application by using the top six significant genes which were identified in the random forest model, by employing the R package “rmda”. Then, the R package “ROC” was used to evaluate the prediction accuracy of the Nomogram. Furthermore, decision curve analysis (35) was conducted to assess the clinical utility of the diagnostic model by using the R package “rmda”. For external validation, we used an independent GEO dataset (GSE142083, n = 106) to validate the performance and robustness of the diagnostic model.

### 2.8 Identification of metabolism-associated genes prognosis model

To construct a metabolism-related prognosis model, the 56 final metabolism-related genes were incorporated into LASSO Cox regression analyses. Kaplan-Meier (KM) curves were constructed to compare the survival differences between the high- and low-risk score groups (36). To estimate the model’s predictive accuracy, the package “ROC” was used to assess the area under the curve (AUC) values of ROC curves for survival state and 1-, 3-, and 5-year survival rates. The RiskScore and other clinical parameters predictions of the 5-year survival rate in the TCGA dataset were also assessed by using ROC curves. Univariate and multivariate Cox regression analyses of clinicopathological features were further conducted to evaluate whether the RiskScore was an independent predictive factor. The GEO dataset (GSE27020, n = 109) was used for validation. The Wilcoxon test was used to analyze the differences in prognostic signature genes’ expression and RiskScore distribution between the two subtypes. ssGSEA was applied to estimate the relative fraction of 13 immune-related functions and 23 immune cells by using the “GSVA” package (27). OncoPredict was used to estimate differences in drug sensitivity between the two groups using the R package “oncoPredict” (37, 38).

### 2.9 Single-cell analysis further validated the subtypes, diagnostic models and prognostic models

To analyze the single-cell RNA sequencing from the GEO database (GSE252490) for laryngeal squamous cell carcinoma (LSCC), we used the packages including “Seurat” and “SingleR” (39) to carry on standard quality control procedures including “PercentageFeatureSet”, “NormalizeData”, “HarmonyIntegration”, “RunPCA”, “FindNeighbors”, “FindClusters”, “RunUMAP”, and “FindAllMarkers” functions. Besides, we used the “SingleR” package and the existing markers from the previous studies (19) to distinguish the cell types. Copy number variation (CNV) scores for epithelial cells were calculated using the “CopyKat” package (40), with B cells as the reference. To understand the biological roles of marker genes within each cluster of malignant epithelial cells, we utilized the “clusterProfiler” and “org.Hs.eg.db” software packages. Additionally, we investigated the developmental paths of various malignant epithelial cell categories using the Monocle2 algorithm (41). Furthermore, we employed the “AddModuleScore” (42) to compute scores related to subtypes, diagnostic model, and prognostic model, which were based on the mean expression levels of genes.

### 2.10 Statistical analysis

All statistical analyses were performed using R software (version 4.3.2 and version 4.4.0). The Wilcoxon test was used to analyze the differences among groups. Survival data were analyzed using the KM curves. In particular, P < 0.05 was considered to be statistically significant.

## 3 Results

### 3.1 Two distinct tumor subtypes in LCA were identified by Consensus clustering

**Figure 1:**
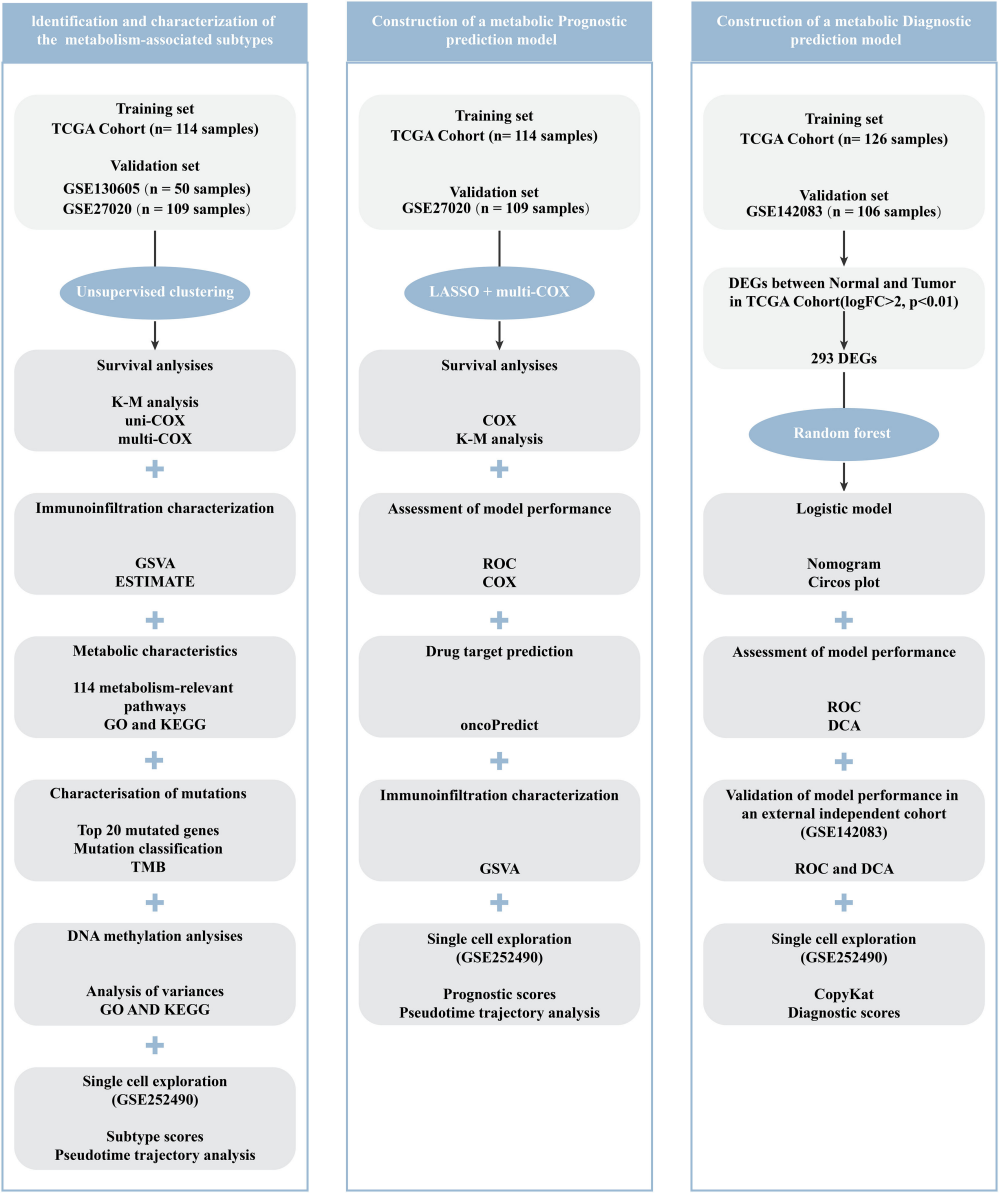
Flow chart of this work.

**Figure 2:**
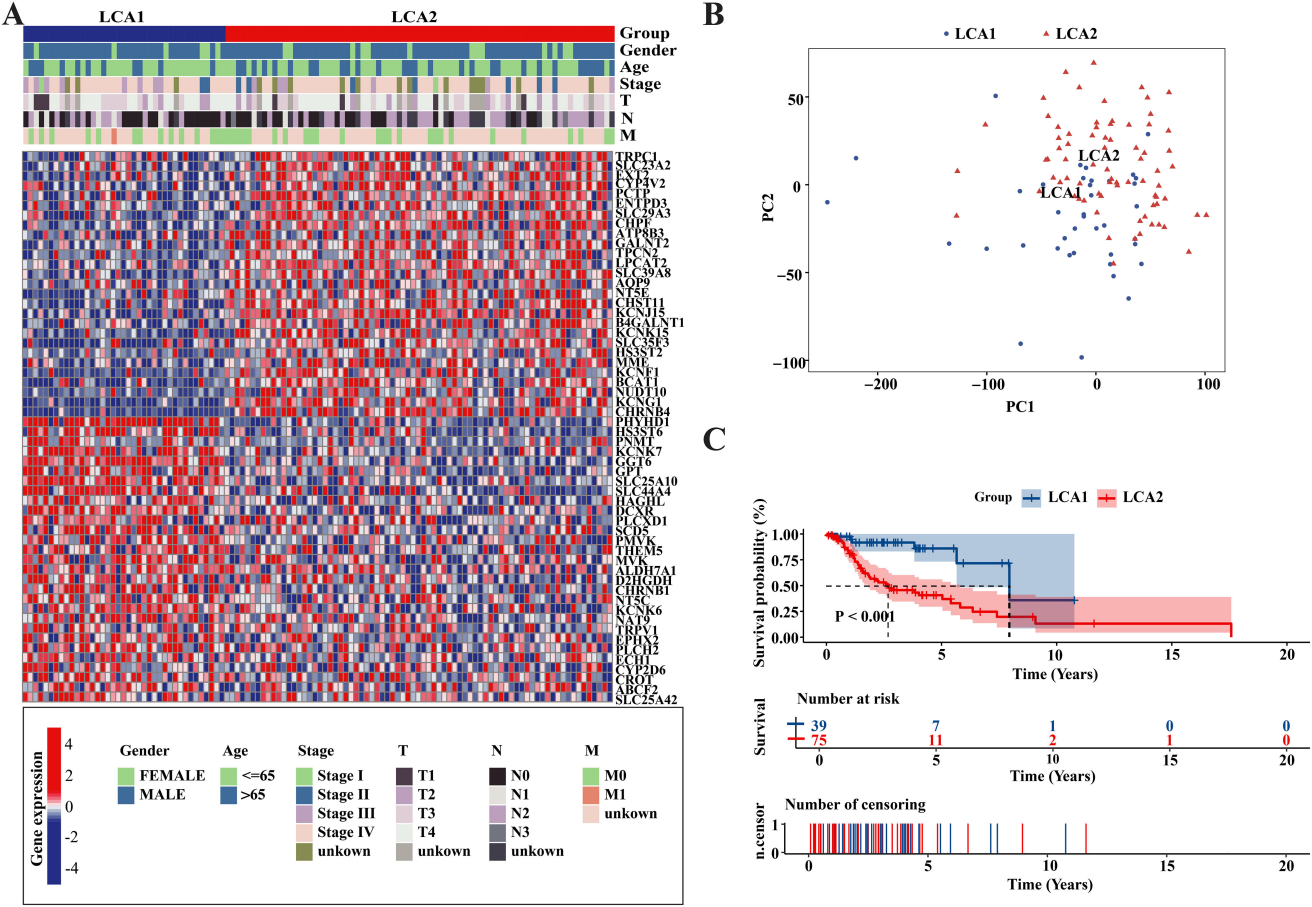
Metabolism-related genes’ profile of LCA defined two subtypes in TCGA cohort. **(A)** Heatmap showed that two metabolic subtypes were defined in TCGA cohort. 56 final candidate genes were shown. Patients were arranged based on the subtypes. Clinical information was also annotated for each patient. **(B)** Principal component analysis (PCA) was applied between the two subtypes among the whole transcriptome data. **(C)** The result of Kaplan-Meier curves indicated that the overall survival time were significantly different between the two subtypes in the TCGA dataset. P value was calculated by the log-rank test between the two subtypes.

**Figure 3:**
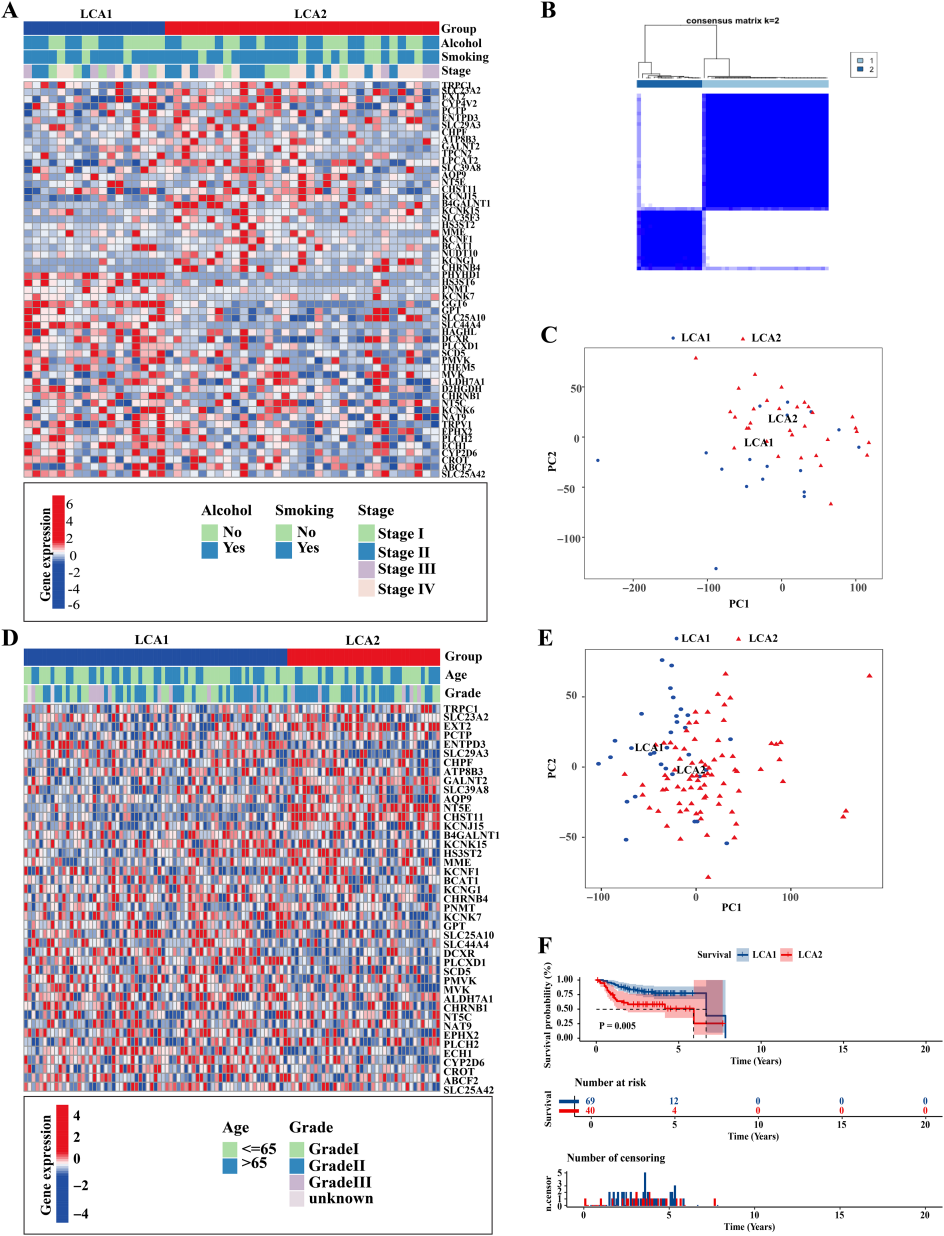
Repeatability verification in other two independent datasets, GSE130605 and GSE27020. **(A, D)** Heatmaps showed that two metabolic subtypes were defined in the GSE130605 and the GSE27020 cohorts, respectively. Clinical information was also annotated for each patient. **(B)** Consensus matrix clustered patients of GSE130605 dataset into 2 clusters. **(C, E)** Principal component analyses (PCA) were applied between the two subtypes by using the whole transcriptome data in both GSE130605 and GSE27020 cohorts. **(F)** The result of Kaplan-Meier curves indicated that the disease-free survival (DFS) was significantly different between the two subtypes in the GSE27020. P value is calculated by the log-rank test between subtypes.

To investigate the metabolic heterogeneity in LCA, we collected 2752 metabolism-related genes from previous studies for clustering analysis (22). The workflow of this study is illustrated in **Figure 1**. Initially, we adopted four study methods: MAD > 0.5, univariate COX regression analysis of survival, consensus clustering analysis, and differential expression analysis of the two subtypes. This process yielded 56 genes and two metabolism-related subtypes including *LCA1* and *LCA2* (**Figure 2A**, **Supplementary Figure S1**). Principal component analysis (PCA) was performed to assess the composition of the two subtypes and confirm the stable expression differences between the two subtypes (**Figure 2B**). Survival analysis revealed that the prognosis of *LCA1* was significantly better than that of *LCA2* (P<0.001) (**Figure 2C**), whereas the clinical characteristics of the two subtypes did not show any significant differences (**Supplementary Table S1**). Finally, we validated the reproducibility of the two subtypes in the two independent datasets including GSE130605 and GSE27020. We identified similar metabolism-related molecular subtypes of LCA in both the GSE130605 (**Figures 3A–C**; **Supplementary Figure S2**), and the GSE27020 datasets (**Figures 3D–F**, **Supplementary Figure S3**) by performing consensus clustering analysis of the final candidate genes and conducting PCA on the entire transcriptome data.

### 3.2 Metabolic profiling of the two subtypes showed significant differences

**Figure 4:**
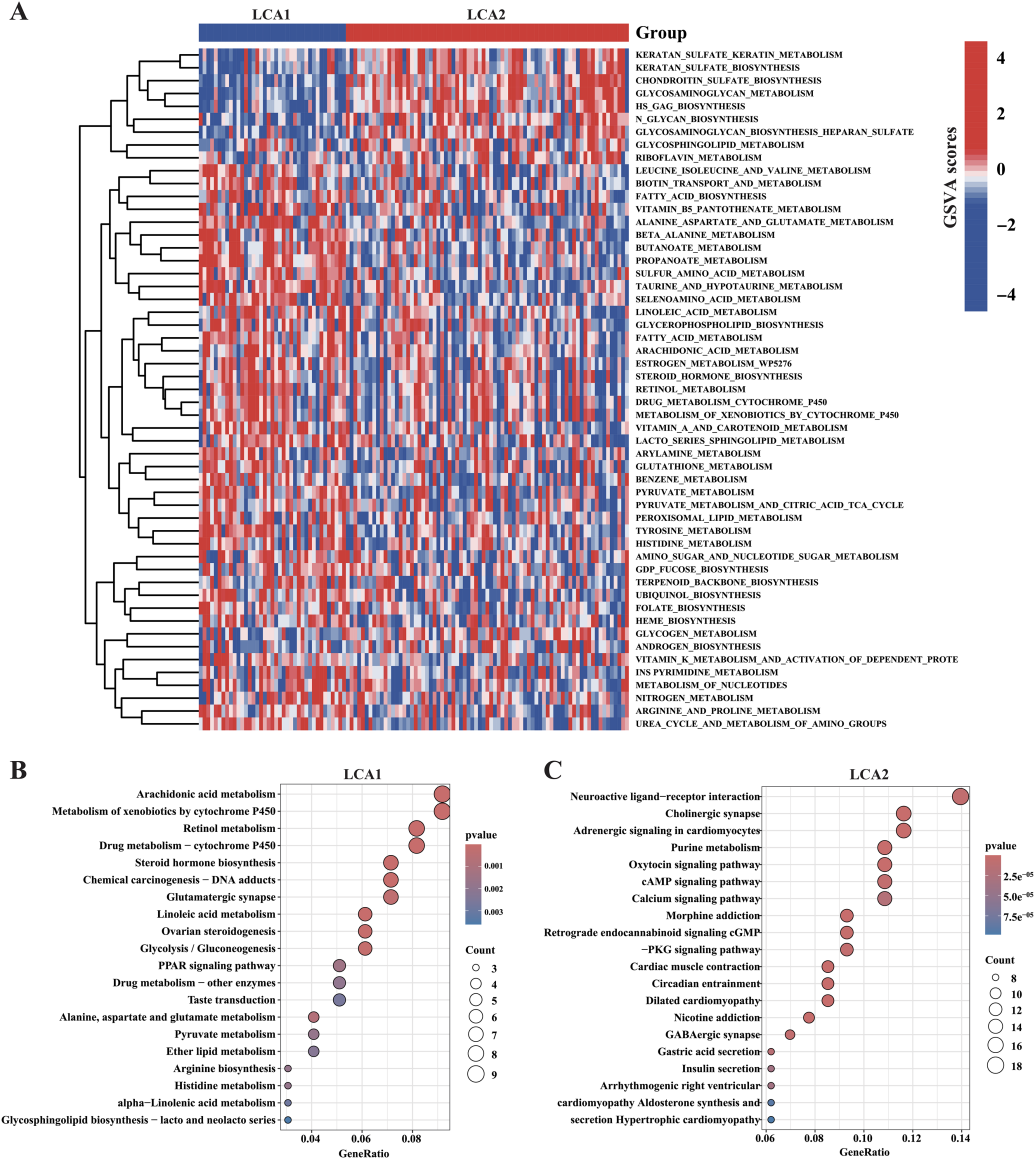
Association between the acquired metabolic subtypes and metabolism-relevant signatures. **(A)** Heatmap showed the differential enrichments of 114 metabolism-related pathways in the TCGA cohorts (the pathways of P < 0.05 were shown in the heatmap, by Wilcon rank-sum test). **(B, C)** The result of KEGG enrichment analysis showed the highly expressed metabolism-relevant genes’ profiles of the two subtypes.

We further investigated the metabolic characteristics of the two subtypes. Initially, the GSVA method was used to calculate the scores of 114 metabolic related pathways’ scores. *LCA1* exhibited 44 specific metabolic signatures, while *LCA2* had nine (**Figure 4A**; **Supplementary Table S2**). Then, this study applied differentially expressed gene (DEGs) analysis to analyze the 2752 metabolism-related genes, and 143 highly expressed genes in *LCA1* and 194 in *LCA2* were identified (Absolute |log2 fold change (FC)| > 1 and Adjusted P-value < 0.05). Both KEGG pathway and GO functional enrichment analyses were used to analyze the highly expressed genes in the two subtypes, respectively (**Figures 4B, C**; **Supplementary Figure S4A**). Finally, the results of the enrichment analyses indicated that *LCA1* had higher levels of metabolism processes compared with *LCA2*, which were related to amino acids, lipids, and vitamins. In contrast, *LCA2* tumors showed few enrichments of metabolic signatures such as glycosylation, which indicated their lower metabolic activities.

### 3.3 LCA2 showed a higher degree of immune infiltration

**Figure 5:**
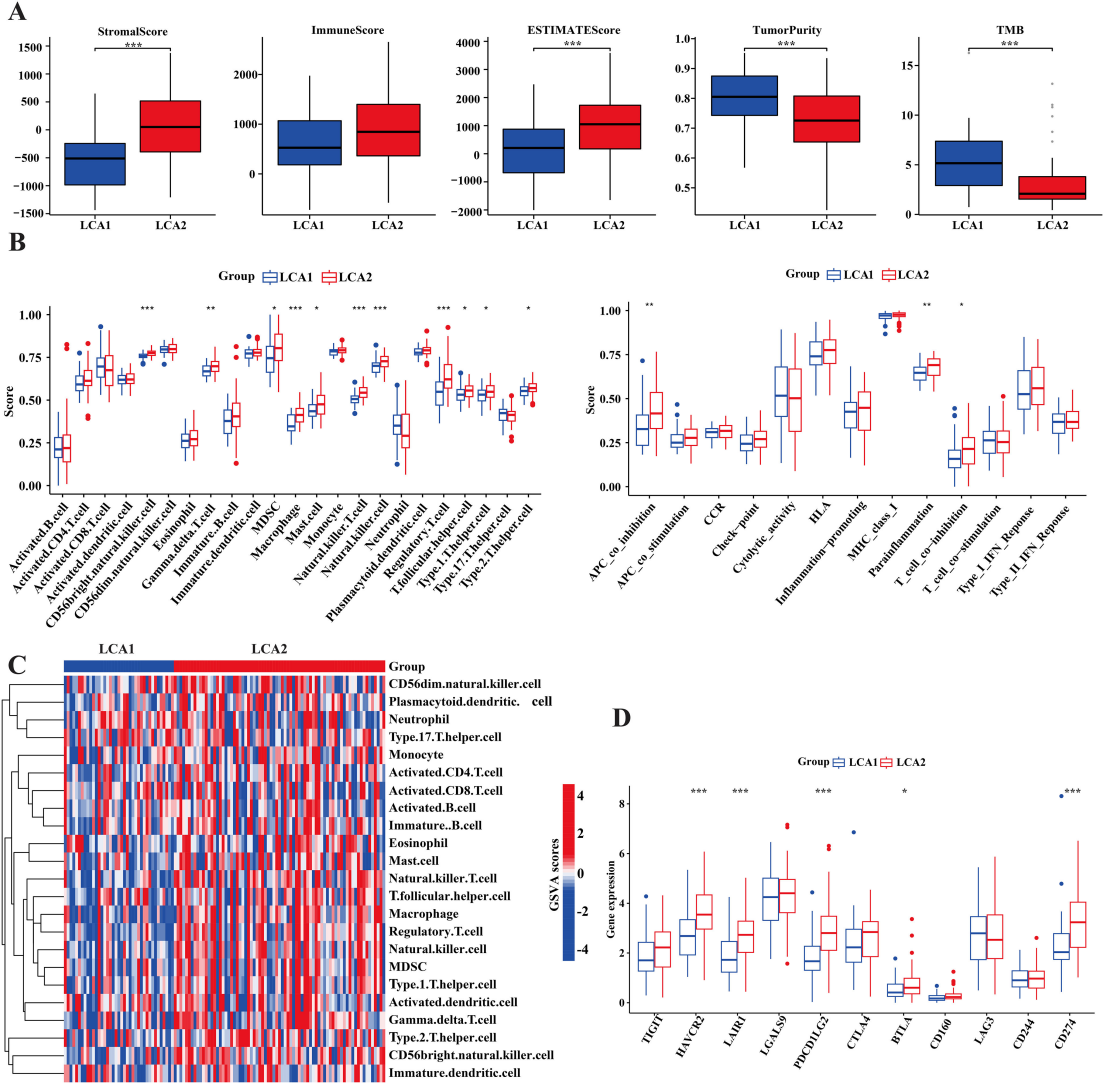
Tumor immune infiltration differences of the two subtypes in TCGA cohort. **(A)** Immune components, stromal components and tumor components were counted by ESTIMATE algorithm, while tumor mutation burden (TMB) was counted by using the R package ‘maftools’. **(B)** Box plot compared the differences of 13 immune-related functions and 28 immune cells between the two subtypes by using the “GSVA” package. **(C)** Heatmap showed the differences of immune-related cells in the two subtypes. **(D)** Box plot compared the 11 immune checkpoint genes’ expression between the two subtypes. (*P < 0.05, **P < 0.01, ***P < 0.001, Wilcon rank-sum test).

It is well established that the amino acid metabolism reprogramming in tumors plays a critical role in controlling the differentiation and function of immune cells (43). To explore the differences in immune infiltration between *LCA1* and *LCA2*, we calculated the ImmuneScore, StromalScore, ESTIMATEScore, and TumorPurity by using the ESTIMATE algorithm. Consequently, *LCA2* had a higher StromalScore and ESTIMATEScore, whereas *LCA1* had a higher TumorPurity (**Figure 5A**). Then, the result of “ssGSEA” analysis showed higher scores for the three immune-related functions and 11 immune cells in *LCA2* (**Figures 5B, C**). Additionally, because T cell and natural killer cell inhibition are known to be important mechanisms for immune escape in cancer, we continued to explore the different expression levels of various immune checkpoint genes between the two subtypes. Consequently, several checkpoint genes showed higher expression levels in *LCA2*, which indicated that a higher level of immunosuppression was observed in this group of *LCA2* (**Figure 5D**).

### 3.4 Nonsense mutations of NSD1 and genome-wide hypomethylation occurred in the LCA1

**Figure 6:**
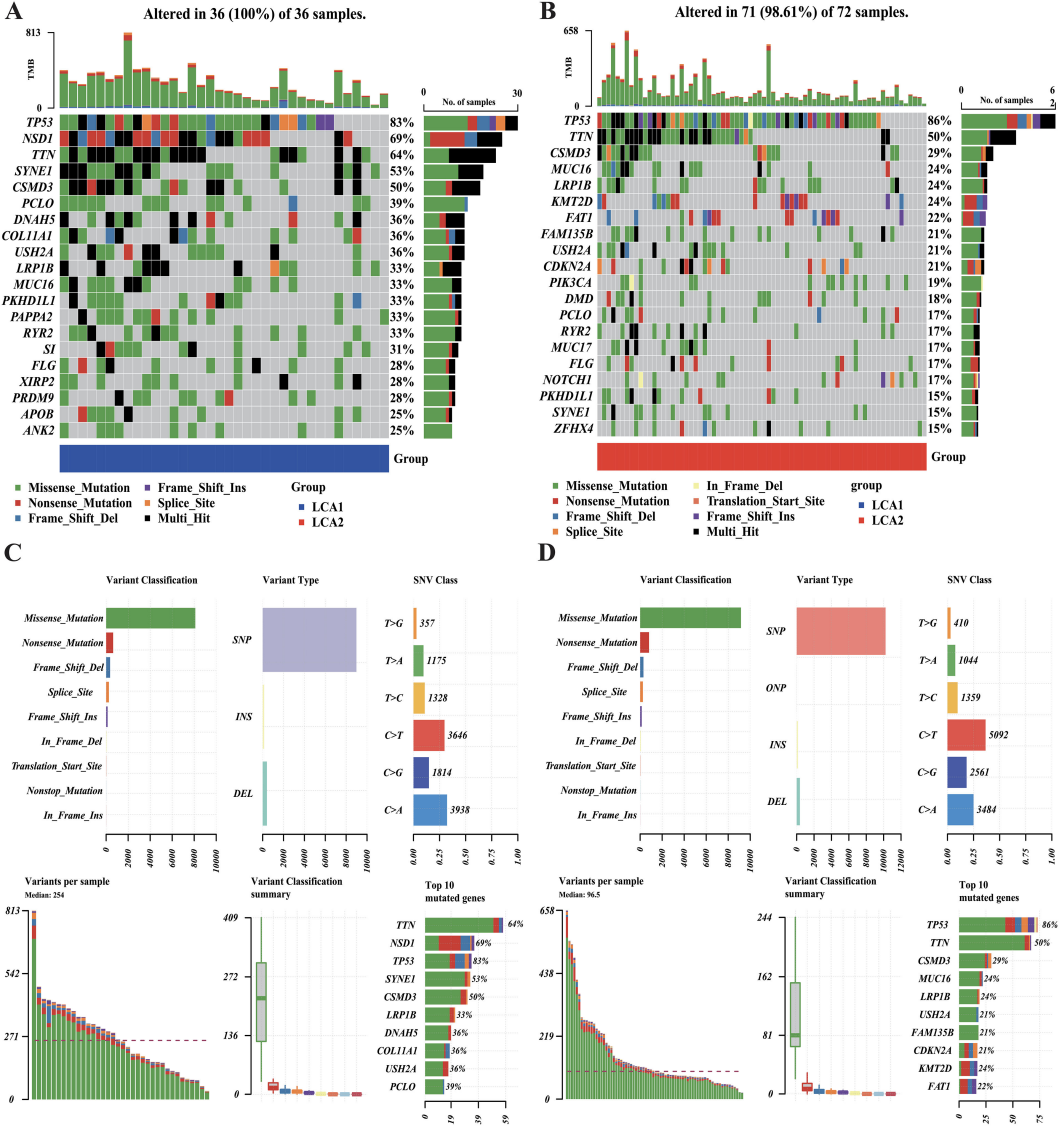
Comparison of the DNA mutations between the metabolic subtypes in TCGA cohort. **(A, B)** Waterfall plots showed the differences in DNA mutations between the two subtypes. The top 20 genes in terms of the frequency of mutation were shown. **(C, D)** The variant classifications, variant types and SNV classes’ summary of two metabolism groups were shown in the plots.

The tumoral genomic landscape has been shown to correlated with antitumor immunity. To investigate whether differences exist in somatic mutation frequencies and patterns between the two subtypes, we compared the somatic variations of the two subtypes and found a higher tumor mutation burden in *LCA1.* In addition, the genes with high mutation frequencies in each subtype are shown in **Figures 6A, B**. Consequently, we found that 11 genes were shared between the two subtypes, whereas each subtype had nine unique high-frequency mutated genes. Notably, the mutation frequency of the histone methyltransferase *NSD1*(69%) in *LCA1* was much higher than that in *LCA2*. However, in terms of variant and SNV classification, the two metabolism-related subtypes showed similar trends (**Figures 6C, D**).

**Figure 7:**
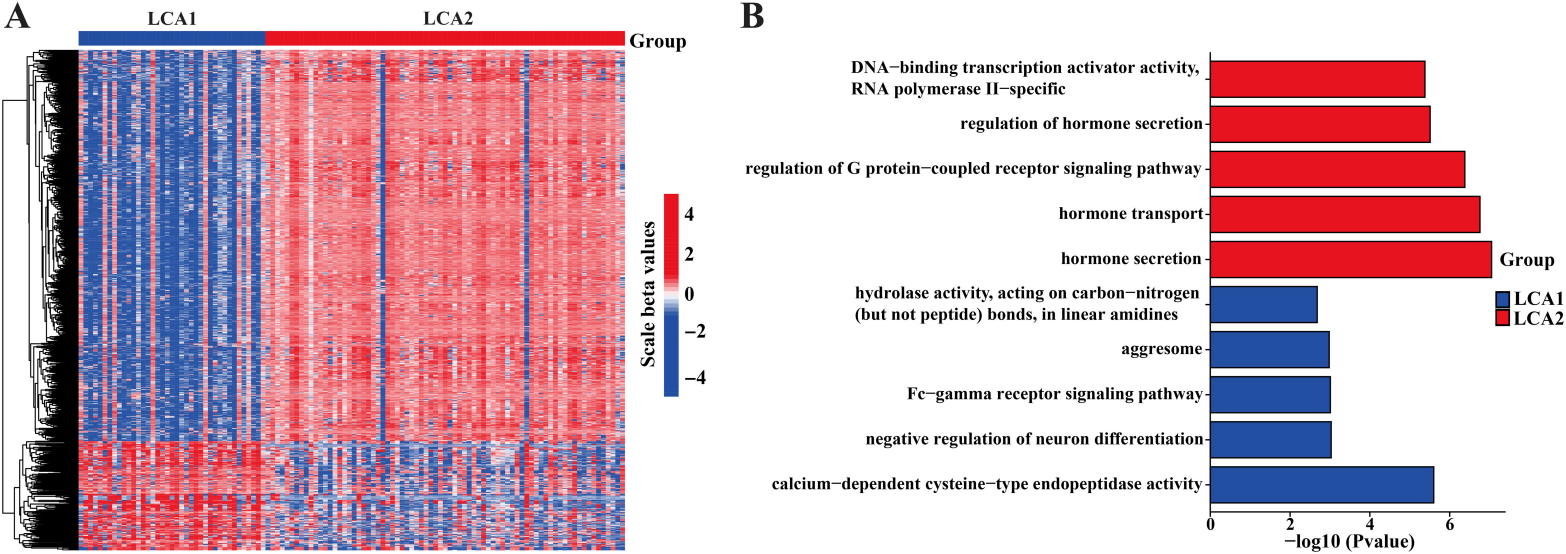
Differences of the DNA methylation between the two subtypes in the TCGA dataset. **(A)** Heatmap showed the differences of the DNA methylation in two metabolism subtypes. **(B)** GO enrichment was used to analyze the highly methylated genes of two subtypes.

The histone methyltransferase *NSD1* is strongly associated with DNA methylation. To further investigate the methylation differences between the two subtypes, we examined the different methylated CpG sites in the TCGA cohort. A total of 1471 CpG probes were significantly differentially methylated between the subtypes (Absolute |log2 fold change (FC)| > 0.25 and Adjusted P-value < 0.01) (**Figure 7A**). Genes with methylation changes in *LCA1* were highly enriched in calcium-dependent cysteine-type endopeptidase activity, negative regulation of neuronal differentiation, Fc-gamma receptor signaling pathway, and aggresome. Differentially methylated genes in *LCA2* were highly enriched in hormone secretion, hormone transport, regulation of the G protein-coupled receptor signaling pathway, regulation of hormone secretion, and DNA-binding transcription activator activity. (**Figure 7B**).

### 3.5 The diagnostic panel, which was derived from machine learning, exhibited an excellent performance

**Figure 8:**
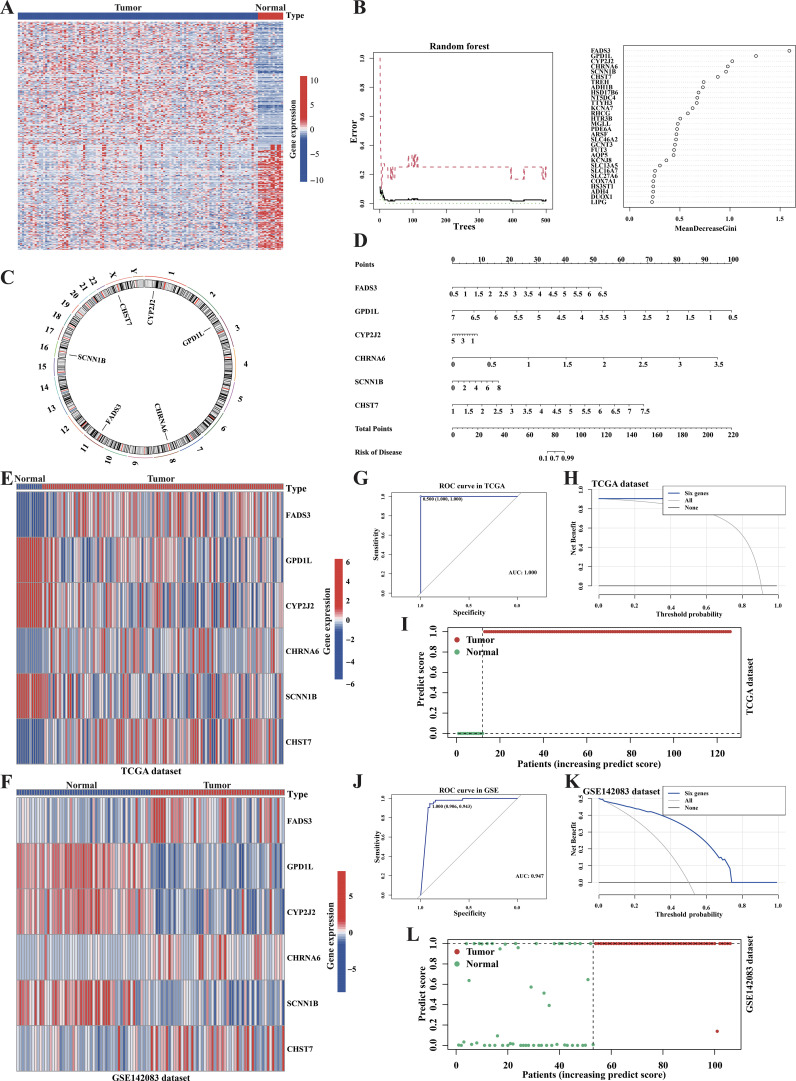
Machine learning identified a metabolism-associated diagnosis prediction model for LCA. **(A)** Heatmap showed the differences of metabolism-related genes’ expressions between normal and tumor in the TCGA cohort (logFC>2, P<0.01). **(B)** Plots showed the result of the Random-forest analysis based on the candidate DEGs. **(C)** Circle plot showed the positions of diagnosis model related genes. **(D)** A nomogram was developed by the diagnosis model’s genes. **(E, F)** Heatmaps showed the diagnosis genes expression between normal and tumor samples in the TCGA dataset and the GSE142083 dataset. **(G, J)** The diagnosis model’s accuracies of prediction in the TCGA dataset and the GSE142083 dataset were assessed by the ROC curves. **(H, K)** Decision curve analysis confirmed the predictive superiority of the diagnostic model. **(I, L)** The plots showed the prediction scores differences between the normal samples and tumor samples.

To develop a robust diagnostic panel, we first identified 273 metabolic genes with significant differential expression between tumor and normal samples through DEGs analysis in the TCGA cohort (Absolute |log2 fold change (FC)| > 2 and Adjusted P-value < 0.01) (**Figure 8A**, **Supplementary Table S3**). Then, machine learning was used to develop a model to predict the clinical status in this study. The random forest model was trained using minimum error regression trees, and the six most essential genes were selected to discriminate the tumor from normal samples (**Figures 8B, C**). Next, a nomogram was developed for clinical use using logistic regression analysis (**Figure 8D**). To further investigate the characteristics of our diagnostic model, we examined the distribution of the model genes in the TCGA samples and the independent dataset GSE142083. The heatmap demonstrated substantial expression differences in the model genes between the tumor and normal samples (**Figures 8E, F**). In order to validate the performance of the diagnostic panel, we calculated the predicted scores of each sample. ROC analysis showed an AUCs of 1 for TCGA and 0.947 for GSE142083, indicating that the model had an excellent diagnostic predictive value (**Figures 8G, J**). We also observed significant differences in the predictive scores between tumor and normal samples (**Figures 8I, L**). Decision Curve Analysis (DCA) plots also confirmed the predictive superiority of our diagnostic model in both TCGA and GSE142083 datasets (**Figures 8H, K**).

### 3.6 The metabolic prognostic model accurately predicted the outcomes of patients with LCA

**Figure 9:**
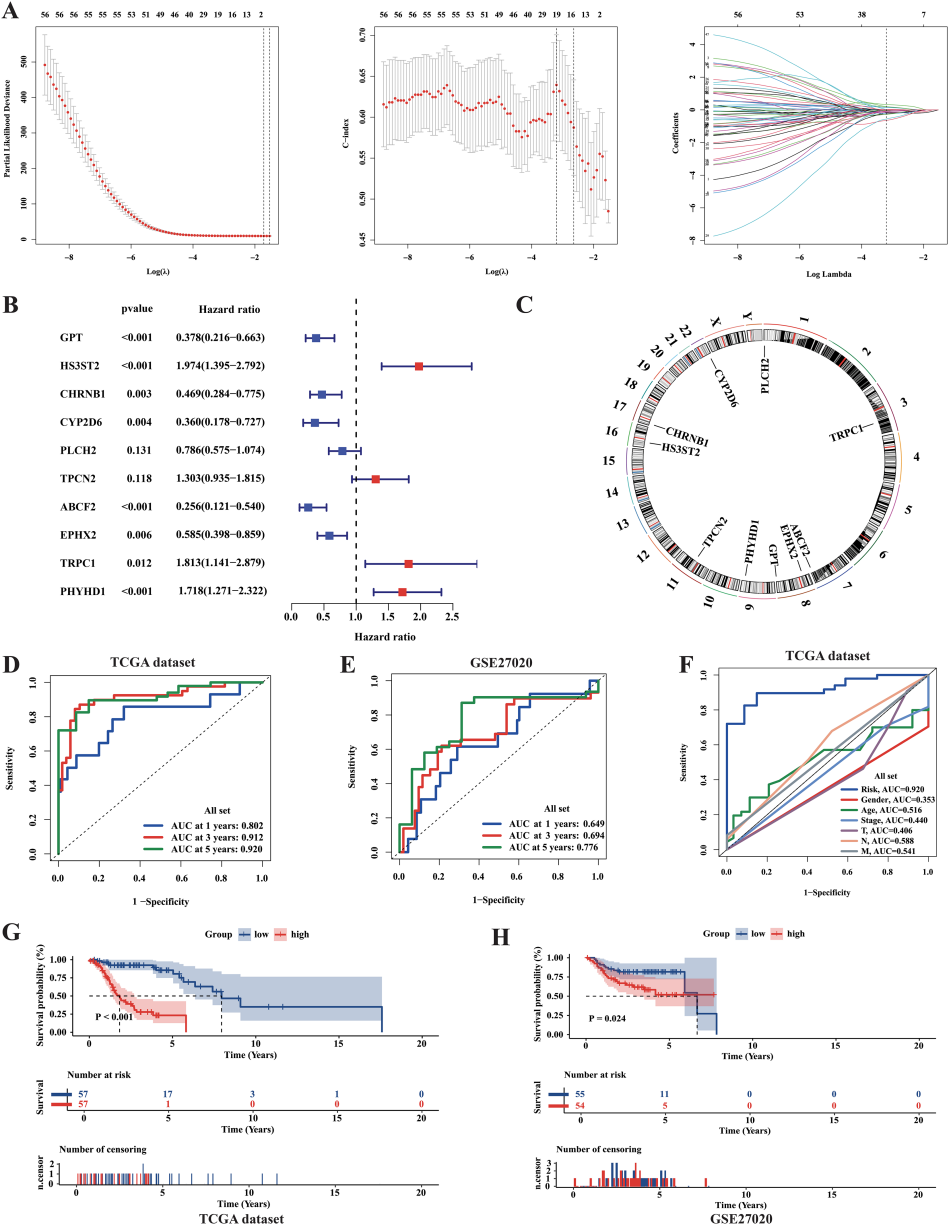
Identification of metabolic prognosis prediction model for LCA. **(A)** LASSO regression analysis based on survival time and survival state was applied among 56 final metabolism-related genes in the TCGA cohort. **(B)** The result of the Multi-COX regression analysis based on survival time and survival state was shown in the plot. **(C)** Circle plot showed the positions of prognosis-model related genes. **(D)** The ROC curves assessed the ability of the RiskScore in predicting survival rates at 1, 3 and 5 years in the TCGA dataset. **(E)** The ROC curves assessed the ability of the RiskScore in predicting survival rates at 1, 3 and 5 years in the GSE27020 dataset. **(F)** The predictive abilities of the RiskScore and other clinical parameters at 5-year survival rate in TCGA dataset were assessed by the ROC curves. **(G, H)** The results of Kaplan-Meier curves showed huge differences in survival state between two risk groups in both TCGA dataset and GSE27020 dataset. P value was calculated by the log-rank test between groups.

As a precise prognosis could enable precise intervention in tumors and help in the clinical treatment of patients, we also attempted to develop a machine-learning-derived prognostic model. First, using the LASSO regression algorithm and multivariate COX regression analysis, we selected ten essential genes, with six acting as protective factors (*GPT, CHRNB1, CYP2D6, PLCH2, ABCF2*, and *EPHX2*) and four as risk factors (*HS3ST2, TPCN2, TRPC1*, and *PHYHD1*) (**Figures 9A–C**). The RiskScore formula is shown in the Additional file1, RiskScore formular. Based on the median of the RiskScore, the TCGA samples were classified into high- and low-risk groups. Next, the result of KM analysis revealed significantly longer survival time in the low-risk group (**Figure 9G**). To assess the accuracy of our model, we performed ROC analysis, yielding AUC values of 0.802, 0.912, and 0.920 for the 1-, 3-, and 5-year survival rates, respectively (**Figure 9D**). The results of the comparative analysis indicated that the RiskScore had the highest predictive ability for 5-year survival compared with other clinical parameters (**Figure 9F**). Moreover, the clinical characteristics did not show significant differences between the two groups, implying that the prognostic model was actually an independent factor for prognosis prediction (**Supplementary Table S4**). In addition, we used the independent dataset GSE27020 from GEO to validate the model. However, owing to the lack of expression data for *TPCN2* and *PHYHD1*, the RiskScores for GSE27020 were not perfectly verified for repeatability. However, the results of the KM analysis still showed significant differences in disease-free survival (DFS) between the groups (**Figure 9H**). Furthermore, ROC analysis for GSE27020 yielded AUC values of 0.649, 0.694, and 0.776 for the 1-, 3-, and 5-year progression-free survival rates, respectively (**Figure 9E**).

**Figure 10:**
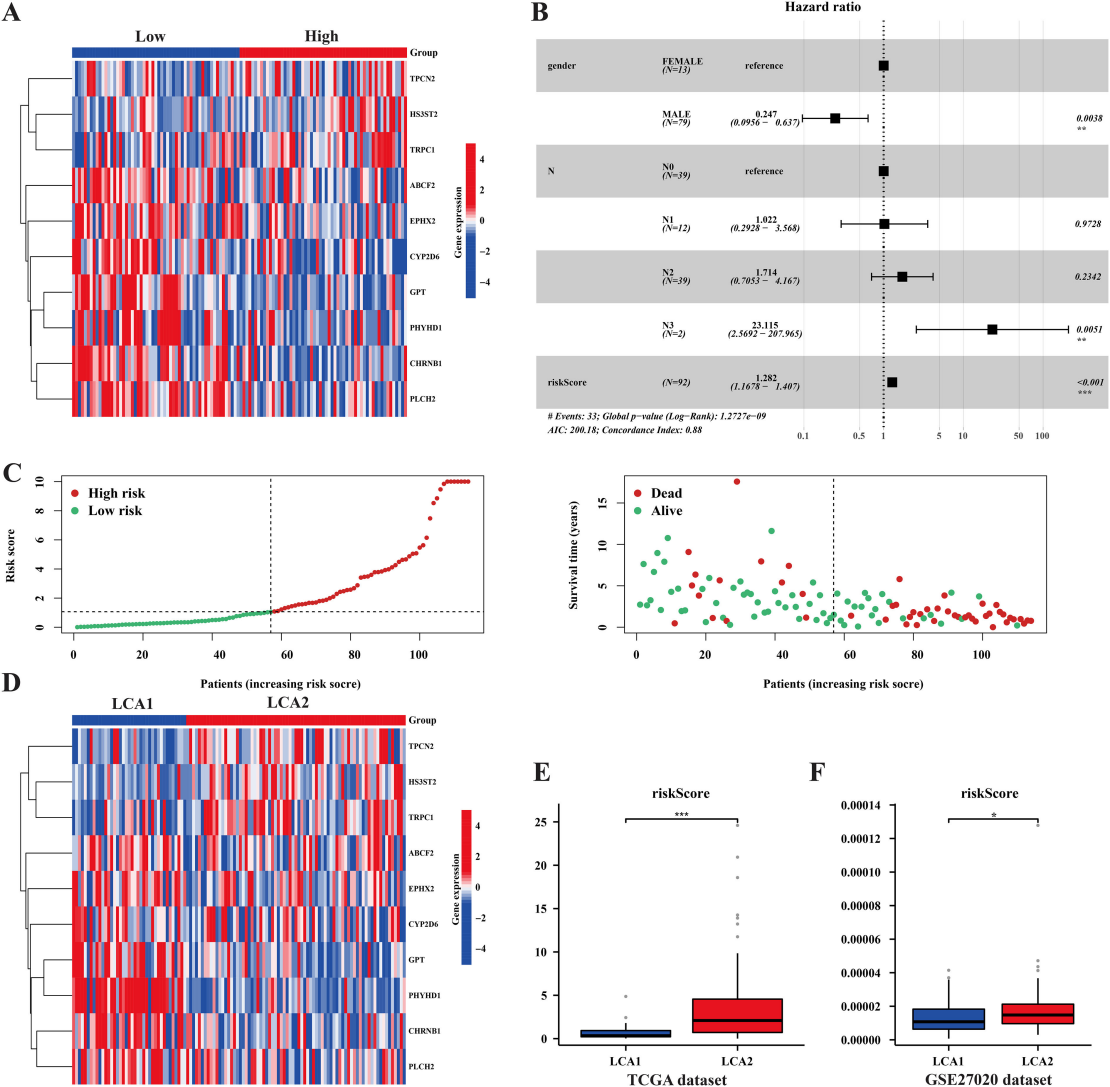
The prognosis model’s characteristics and its relationships with the subtypes. **(A, D)** Heatmaps showed the prognosis related genes’ expression among risk-groups and metabolism subtypes. **(B)** Forest plot of univariate and multivariate cox regression analysis exhibited the relationship between RiskScore and overall survival in LCA patients. **(C)** The plots showed the distribution differences of Riskscore between risk-groups. **(E, F)** The plots showed the distribution differences of the RiskScore between the two subtypes in both the TCGA dataset and the GSE27020 dataset. (*P < 0.05, **P < 0.01, ***P < 0.001, Wilcon rank-sum test).

To better investigate the characteristics of our prognostic model, we examined the distribution of the RiskScore in TCGA samples (**Figures 10A–D**). Additionally, univariate and multivariate Cox regression analyses were performed to assess the prognostic significance of the variable factors, including sex, age, stage, Tumor size (T), Nodal status (N), and RiskScore. As a result, Sex, N, and RiskScore were identified as significant prognostic factors (**Figure 10B**, **Supplementary Figure S4B**). Notably, the distribution of the RiskScore between the two metabolic subtypes showed significant differences in both the TCGA and GSE27020 datasets (**Figures 10E, F**).

We also explored the differences in immune cell infiltration between the high- and low-risk groups. Several conclusions were drawn. Firstly, the result of ssGSEA analysis indicated that the scores of 5 immune cell scores and 1 immune-related function were higher in the high-risk group (**Supplementary Figures S5A–C**). Furthermore, correlation analysis between immune cells and the RiskScore was conducted using TIMER, XCELL, ABS, CIBERSORT-ABS, QUANTISEQ, MCPCOUNTER, EPIC, and CIBERSORT, with significant results (P < 0.05) displayed (**Supplementary Figure S5D**). Finally, OncoPredict was utilized to forecast drug sensitivity in the LCA. Differences in drug sensitivity differences with P < 0.001 between the two groups were presented in **Supplementary Figure S5E**.

### 3.7 Subtype, diagnostic, and prognostic related genes were analyzed in the single-cell sequencing

**Figure 11:**
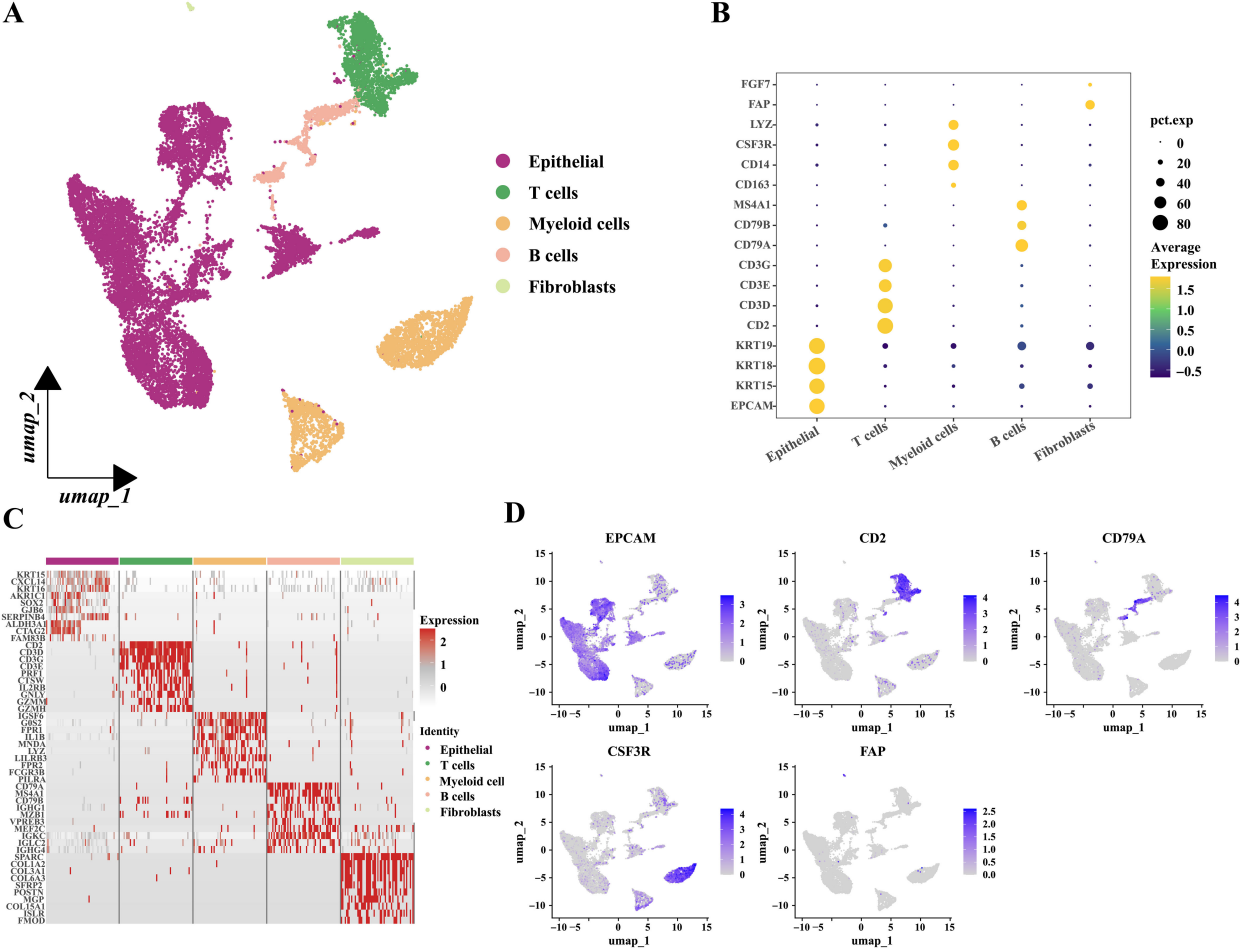
Grouping and identification of cells in laryngeal cancer tissues. **(A)** Umap plot showed the distribution of the five types of cells. **(B)** Bubble plot showed the marker genes of different cell types. **(C)** Heatmap showed the relative expression of the top ten most differentially expressed genes in each cell type. (Epithelial; T cells; Myeloid cells; B cells and Fibroblasts.) **(D)** Feature plots presented the typical marker genes’ expressions for each cell type.

**Figure 12:**
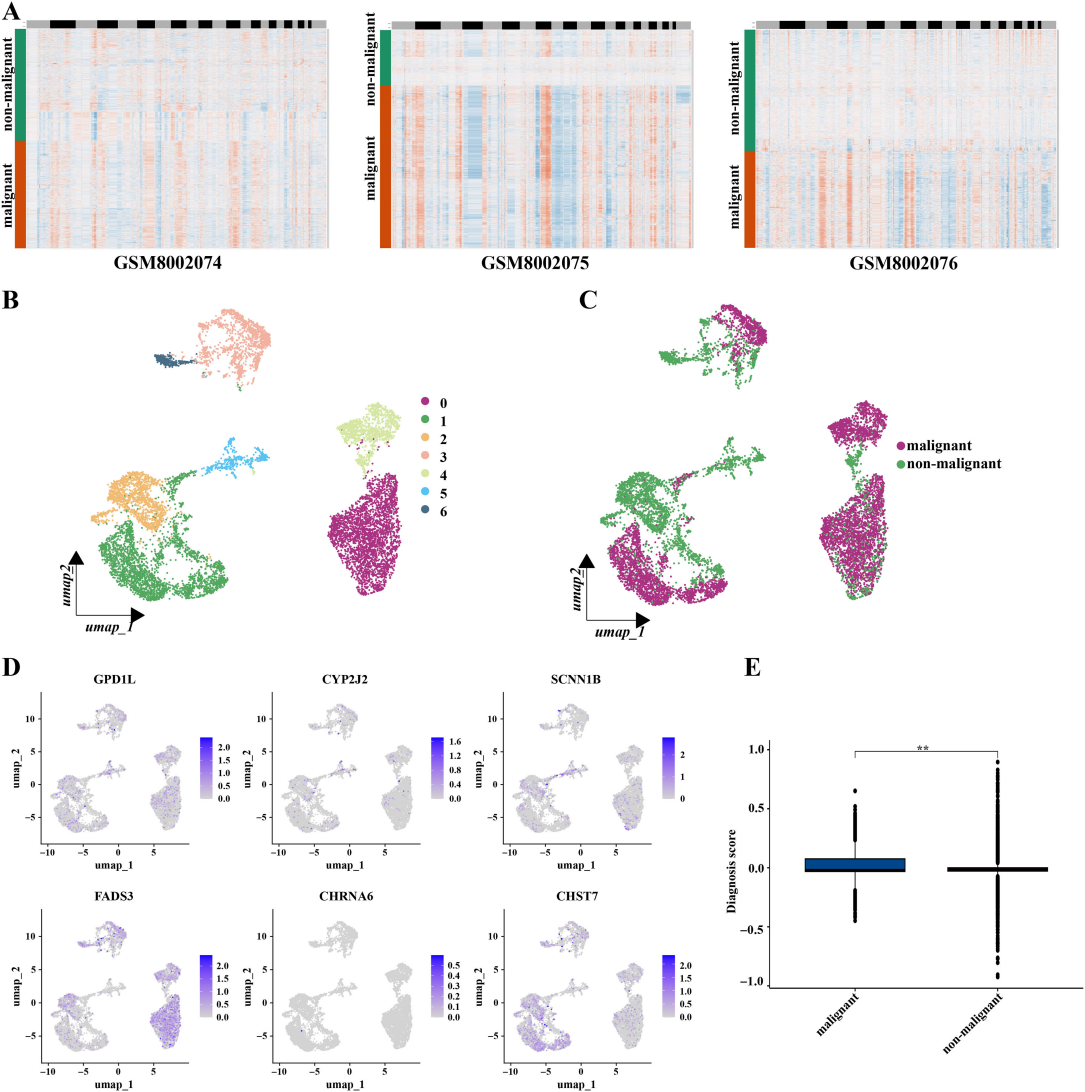
Diagnostic related genes expressions’ analysis based on single-cell sequencing. **(A)** Chromosomal landscape of CNVs distinguished malignant epithelial-derived cells from non-malignant epithelial-derived cells in different samples. The references were B cells; chromosomal amplifications were shown in red and deletions in blue. **(B)** Umap plots showed the distribution of the seven epithelia-derived cell subclusters. **(C)** Umap plots showed the distribution of the malignant epithelia -derived cell and the non-malignant epithelia-derived cells. **(D)** Feature plots presented the diagnosis related genes’ expressions for epithelial-derived cells. **(E)** The diagnosis scores’ distribution between the malignant epithelia-derived cell and the non-malignant epithelia-derived cells showed huge differences. (*P < 0.05, **P < 0.01, ***P < 0.001, Wilcon rank-sum test).

To explore gene expression related to metabolic subtypes, diagnostic and prognostic models at the single-cell sequencing level, we analyzed a single-cell dataset comprising three samples, GSE142083. After a series of quality control procedures, 16,452 cells were selected for further analysis. Initially, using an appropriate resolution, the cells were divided into 14 clusters and annotated with five cell types (**Figure 11A**; **Supplementary Figure S6A**). Marker genes for the five cell types are shown in (**Figures 11B, D**). The most differentially expressed genes in each cell type are shown in **Figure 11C**. Next, epithelial-derived cells were extracted for further analysis. Epithelial-derived cells were classified into six clusters using an appropriate resolution (**Figure 12B**). Duplicates were identified and removed from the six clusters (**Supplementary Figures S6B, C**). Furthermore, the “CopyKat” algorithm was then applied to each sample individually, which classified epithelial-derived cells into malignant and non-malignant categories (**Figures 12A, C**). Then, we examined the distribution of diagnostic model-related genes in epithelial-derived cells (**Figure 12D**) and scored these cells by using the “AddModuleScore” function. Notably, the diagnostic scores for malignant and non-malignant epithelial cells were significantly different (**Figure 12E**).

**Figure 13:**
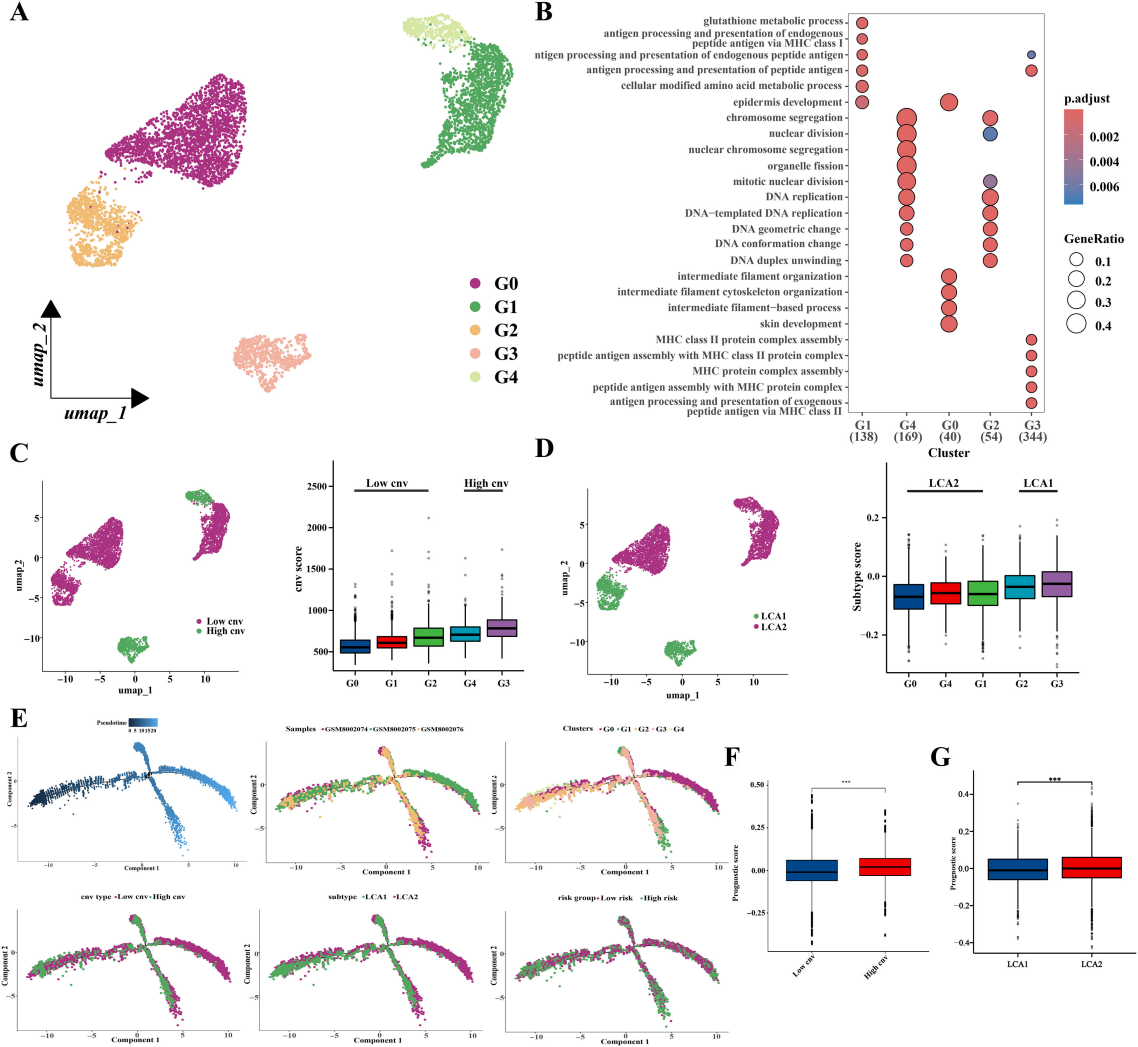
Prognostic related genes expressions’ analysis based on single-cell sequencing. **(A)** Location distribution of different types of malignant cells was shown in umap. **(B)** GO analysis results showed pathway activation differences among the five cell subclusters. **(C)** Umap and box plots showed the distribution of the low-cnv cells and the high-cnv cells. **(D)** Umap and box plots showed the distribution of the *LCA1* cells and the *LCA2* cells. **(E)** Pseudotime analyses of malignant epithelia-derived cells. **(F, G)** The prognostic scores’ distribution between both the cnv groups and the two subtypes showed huge differences. (*P < 0.05, **P < 0.01, ***P < 0.001, Wilcon rank-sum test).

In addition, malignant epithelial cells (6451 cells) were then extracted and classified into five clusters using an appropriate resolution (**Figure 13A**). Highly expressed genes in the five clusters were analyzed by using Gene Ontology (GO) enrichment (**Figure 13B**). Next, the five clusters were categorized into high (G3 and G4) and low (G0, G1, and G2) variant groups based on CNV scores, which were obtained from the “CopyKat” algorithm (**Figure 13C**). Furthermore, we also investigated the distribution of genes related to prognostic risk models in malignant epithelial cells and scored the malignant epithelial cells to obtain the prognostic scores by using the ‘AddModuleScore’ function (**Supplementary Figure S6D**). The “AddModuleScore” function was used to score genes associated with subtyping (**Supplementary Table S5**). Cells were scored based on the characteristic genes that were highly expressed in the two subtypes, and subsequently were divided into *LCA1* (G2 and G3) and *LCA2* (G0, G1, and G4) groups (**Figure 13D**). The relationships between CNV groups and prognostic scores was also investigated. Significant differences in the prognostic scores were observed between the high- and low-variant groups (**Figure 13F**). Additionally, notable discrepancies in prognostic scores were observed between the metabolic subtypes (**Figure 13G**). Finally, a pseudotime trajectory analysis using “Monocle 2” was performed to explore the underlying evolution of epithelial cells with diverse CNV scores and prognostic scores (**Figure 13E**).

## 4 Discussion

Currently, the classification of LCA mainly relies on the sites of its occurrence and the types of pathology, but remains inadequate for treatment guidance (8). In addition, the lack of diagnostic and therapeutic biomarkers has led to delayed late-stage diagnosis and unimproved 5-year survival rates in LCA. To enrich the classification method, we classified LCA into two subtypes based on the 56 metabolism-related genes. And we further explored the differences in clinical and metabolic characteristics, immune infiltration, DNA mutations, DNA methylation, transcriptional data, and single-cell data between the two subtypes. In addition, we established highly reliable diagnostic and prognostic models using machine learning methods. These findings were validated to ensure robustness by using three independent bulk RNA datasets and a single-cell RNA dataset. In conclusion, our findings extended the molecular subtyping of LCA and deepen our understanding of metabolic heterogeneity within this tumor.

There is a consensus that the metabolic reprogramming of tumors leads to distinct metabolic profiles compared to normal tissues (13, 14), however, the metabolism of different tumors and different sections of the same tumor is also heterogeneous (44). Moreover, the metabolic preferences of tumors change dynamically during cancers progression (45). Tumor metabolic heterogeneity is a prominent feature of tumor development and has an important impact on the effectiveness of treatment and prognosis (46). A previous study demonstrated that metabolic heterogeneity in lung cancer tissues may lead to tumor progression and drug resistance (47). In our study, the enrichment of metabolic pathways between the LCA subtypes also showed heterogeneity; *LCA1* was enriched in the amino acid, lipid, and vitamin pathways, whereas *LCA2* was mainly enriched in glycosylation. Glycosylation has been shown to promote tumor invasion (48), suggesting that the therapies targeting glycosylation-related metabolic pathways could be explored for *LCA2*. Meanwhile, the degree of metabolic pathway abnormality was significantly higher in the *LCA2* group. Previous studies have shown that a high degree of metabolic abnormality usually leads to a shorter survival time or resistance to anti-tumor treatments, including chemotherapy, radiotherapy, targeted therapies, and immunotherapy (49).

Although immunotherapy has been widely adopted for cancer treatment (50), progress in LCA immunotherapy has been limited. Some drugs, including cetuximab, pembrolizumab, and nivolumab, have been just used in combination therapy after surgery for advanced LCA (7). The analysis of immune infiltration across the subtypes of *LCA1* and *LCA2* in our study revealed that the infiltration of immune cells, ESTIMATE scores, and certain immune functions (APC_co_inhibition, Parainflammation, T_cell_co_inhibition) were significantly higher in *LCA2*. In addition, multiple immune checkpoint genes (*HAVCR2, LAIR1, PDCD1LG2, BTLA, CD274*) were expressed at higher levels in *LCA2*, suggesting that immunotherapy targeting these inhibitory sites (51) may be effective in this group.

The two subtypes also displayed different patterns of DNA changes. Notably, as a transcriptional regulatory protein with histone methyltransferase activity, *NSD1* exhibits a markedly higher mutation frequency in *LCA1*. Moreover, *NSD1* is primarily involved in processes such as methylation, transcriptional regulation, DNA binding, nuclear receptor binding, and histone modification. As nonsense mutations predominated in *NSD1* variants within *LCA1*, the expression of *NSD1* appeared to be a significantly reduced in *LCA1* (**Supplementary Figure S4C**). This may account for the reduced methylation level observed in *LCA1*. Similarly, a previous study indicated that inactivating mutations in *NSD1* and *NSD2* in LCA are associated with a favorable prognosis (52). Furthermore, inactivating mutations in *NSD1* can inhibit tumor growth, decrease methylation, and reduce immune infiltration (53, 54). All these observations are aligned with our observations. Hence, *NSD1* has a great potential as a key molecule in the classification, prognosis estimation, and targeted treatment of LCA, warranting further explorations.

Indeed, these two isoforms really showed significant differences in their methylation levels. Methylation plays a role in gene silencing, X-chromosome inactivation, and genome stability (55, 56). Moreover, immune infiltration and function are also closely related to methylation levels (57). Up to now, methylation inhibitors are used to treat certain hematological malignancies. These DNA methylation inhibitors can mediate alterations in immune cells functions associated with acquired immunity (58). Typically, cancers exhibit genome-wide hypomethylation and site-specific hypermethylation, and the interactions between genome-wide hypomethylation and site-specific hypermethylation have something to do with the epigenetic and metabolic reprogramming (59). In the present study, *LCA1* exhibited lower levels of methylation, lower immune infiltration, and higher metabolic enrichment, which may be significantly associated with high-frequency nonsense mutations of *NSD1*. Furthermore, the results of the enrichment analysis of methylated sites also revealed the significant differences between the two subtypes: *LCA2* methylated sites were enriched in DNA transcription and hormone-related pathways, particularly in insulin secretion. Hormone secretion and transport have been better explored in some hormone-dependent tumors, such as breast and prostate cancers, and hormone therapies have been employed in their treatment (60, 61). Meanwhile, insulin-related traits are also thought to be strongly associated with cancer development (62, 63). Therefore, the relationship between LCA and insulin-related features requires further investigation.

Patients with LCA often experience a poor quality of life due to the late-stage diagnosis (8). Thus, there is an urgent need to develop non-invasive early screening methods to enhance the precision of medical strategies. Over the past several years, researchers have utilized machine learning algorithms to uncover the hidden relationships between multi-omics data and disease conditions, as well as to develop predictive models (64, 65). In our study, the diagnostic model we had established by machine learning with six signature genes could effectively distinguished tumor samples from normal samples. This model was validated using the independent dataset GSE142083 to ensure its generalizability. The results of the ROC analysis and DCA further confirmed the predictive superiority of the diagnostic model, demonstrating its high clinical utility. On the other hand, the prognostic model with 10 signature genes established by machine learning could effectively predict the prognosis of LCA and was validated using an independent dataset. Previous studies, such as those using *GPT* and *SMS* as prognostic risk models (AUC=0.748, 0.823, and 0.781 for 1-, 3-, and 5-year survival, respectively) (66), and a study using *TMEM2, DACT1, STMN2, GPR173* as prognostic risk models (AUC=0.814, 0.859, and 0.782 for 1-, 3-, and 5-year survival, respectively) (67) also established risk models for LCA. Compared to their models, our model (AUC=0.802, 0.912, and 0.920 for 1-, 3-, and 5-year survival, respectively) showed relatively better predictive accuracy. In the future studies, clinical participants and the improved machine learning algorithms should be utilized to further validate and refine the two models for clinical transformation.

Single-cell sequencing analysis has opened a new era in the exploration of the tumor cell heterogeneity and the tumor microenvironment infiltration, and has been applied to explore cell heterogeneity, immune microenvironment, and drug resistance mechanisms of various types of malignancies (68, 69). In this study, we used scRNA-seq analyses to validate the expression of genes related to metabolic subtypes as well as diagnostic, and prognostic models. The “copycat” was used to distinguish malignant from non-malignant epithelial cells and to obtain CNV scores. Diagnostic scores were calculated for all epithelial-derived cells and were significantly higher for malignant epithelial-derived cells. Malignant epithelial cells were classified into *LCA1* and *LCA2* groups based on the results of the metabolic subtype-related gene analysis. Prognostic risk scores were calculated for malignant cells. It was found that significantly higher prognostic scores were observed in the *LCA2* group. These findings were consistent with the results observed in the bulk RNA datasets, which further confirmed the robustness of the metabolic subtypes as well as the diagnostic and prognostic models.

This study had several limitations. First, the large number of subtype-determining genes, along with their associated high costs, poses a challenge for clinical applicability. Second, although we included a substantial number of patients from both the microarray and RNA-seq platforms, which suggests that our conclusions may be highly reliable and robust without platform bias, the outcomes still require further validation in a prospective study owing to the retrospective nature of the current research. Third, additional experimental studies and validation of the signatures of clinical specimens are necessary for future researches.

## 5 Conclusions

In conclusion, metabolic reprogramming significantly influences tumor growth, progression, and the tumor microenvironment. Despite growing attention to therapeutic approaches targeting tumor metabolism, the diversity and heterogeneity of tumor metabolism pose challenges to metabolic therapy. A comprehensive exploration of metabolic pathways and mechanisms across various tumors is essential to advance metabolic therapies. In this study, we subtyped LCA based on metabolic factors and examined immune infiltration, DNA mutations, methylation, and single-cell data, which laid the foundation for future subclassifications in diagnosis and treatment. Using machine learning, we developed metabolism-related diagnostic and prognostic models with excellent performances. Our work enhances the understanding of patients with LCA classifications, facilitating the development of LCA early detection and shedding light on the precision treatments of LCA.

## Data Availability

Publicly available datasets were analyzed in this study. The data can be found at TCGA (https://portal.gdc.cancer.gov/, accession information: TCGA-HNSC) and GEO (https://www.ncbi.nlm.nih.gov/geo, accession numbers: GSE252490, GSE130605, GSE27020, GSE142083).
