# Supplementary material for: Metabolic profiles in laryngeal cancer defined two distinct molecular subtypes with divergent prognoses
Source: Front Immunol. 2025 May 22;16:1512502. doi: 10.3389/fimmu.2025.1512502 (PMC12137314; doi:10.3389/fimmu.2025.1512502)
Supplement: Supplementary file 1 [file DataSheet1.pdf]

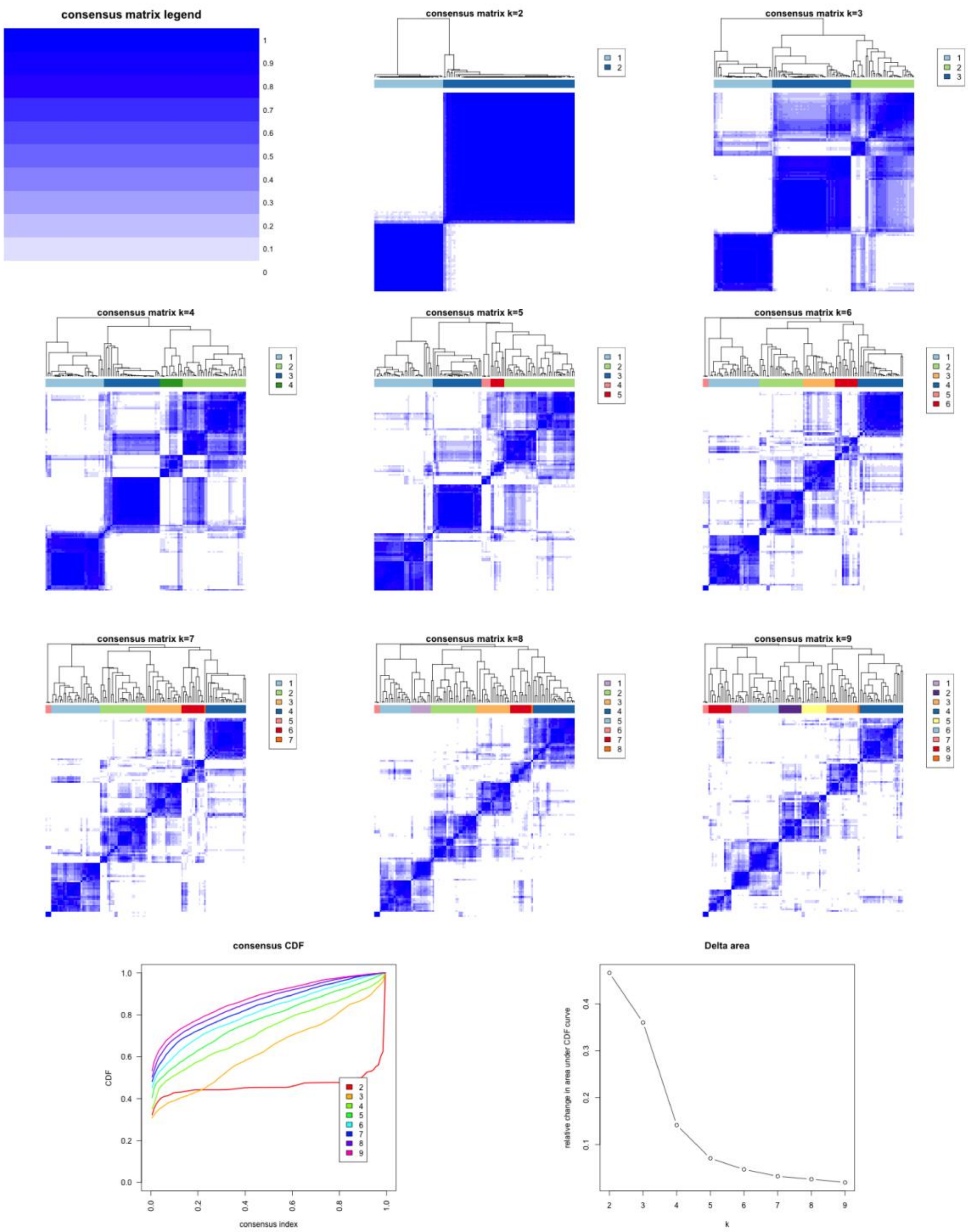

Fig. S1 Consensus matrix of NMF clustering for k = 2–9 and cophenetic correlation coefficient under corresponding k values in TCGA cohort.

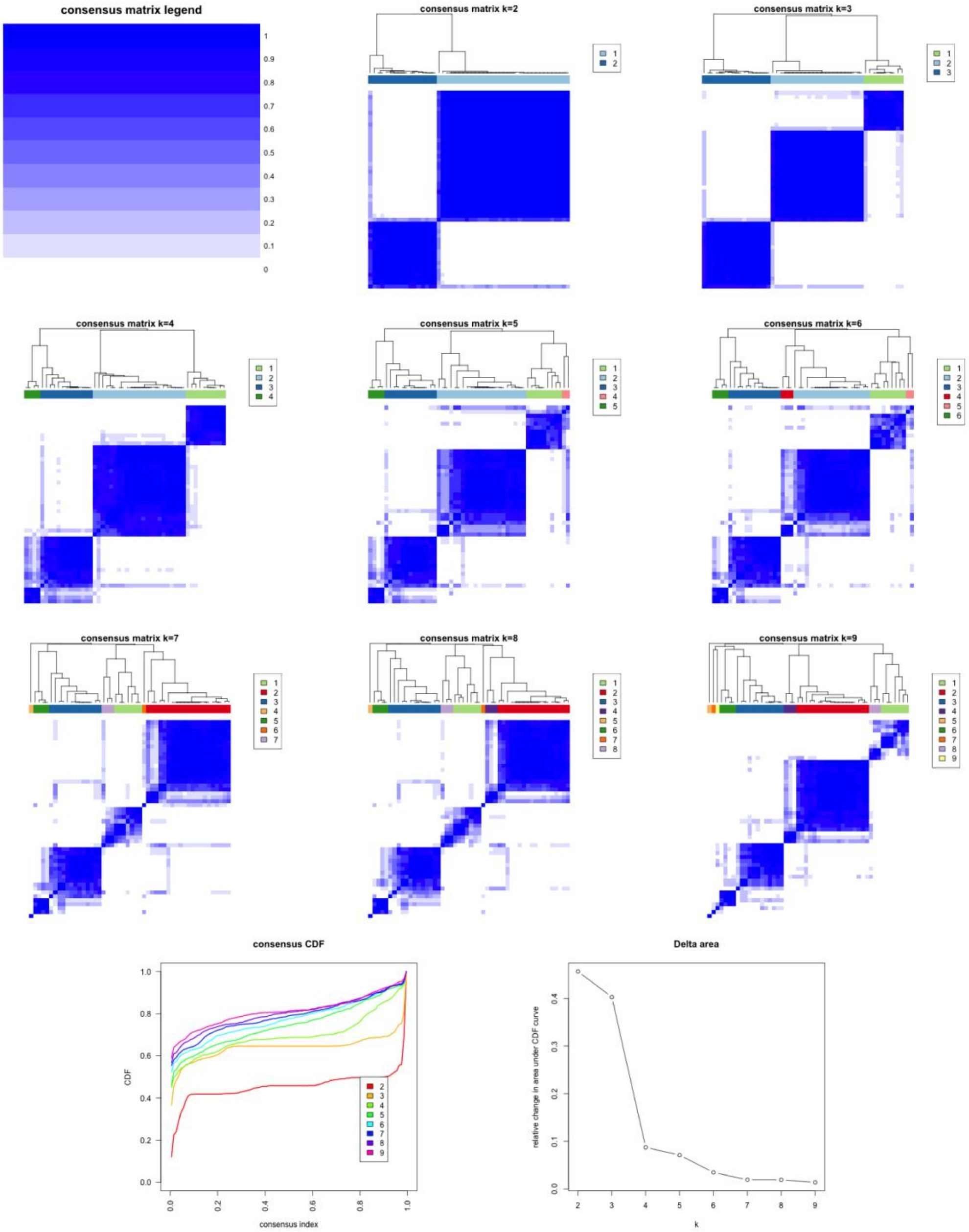

Fig. S2 Consensus matrix of NMF clustering for k = 2–9 and cophenetic correlation coefficient under corresponding k values in GSE130605 cohort.

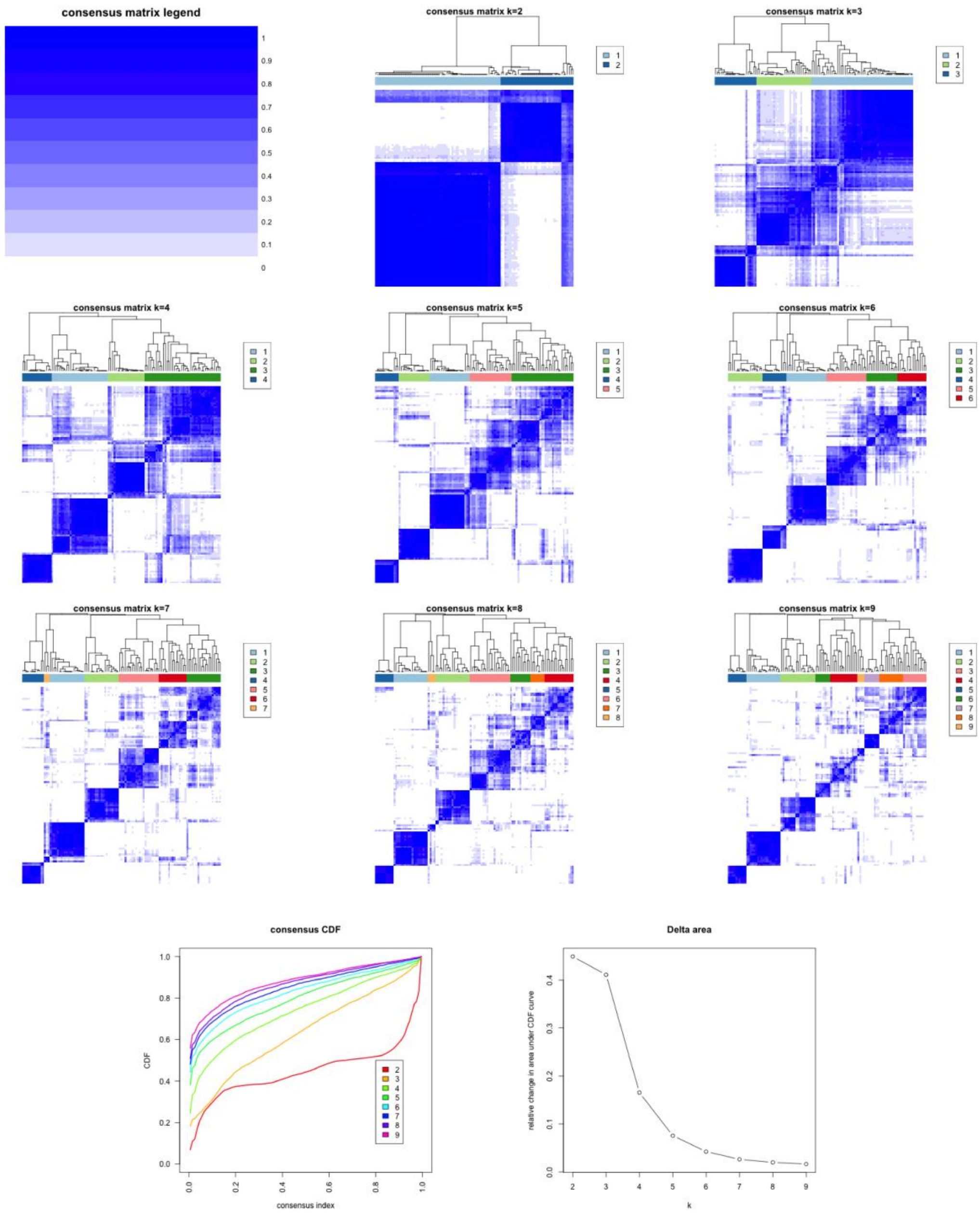

Fig. S3 Consensus matrix of NMF clustering for k = 2–9 and cophenetic correlation coefficient under corresponding k values in GSE27020 cohort.

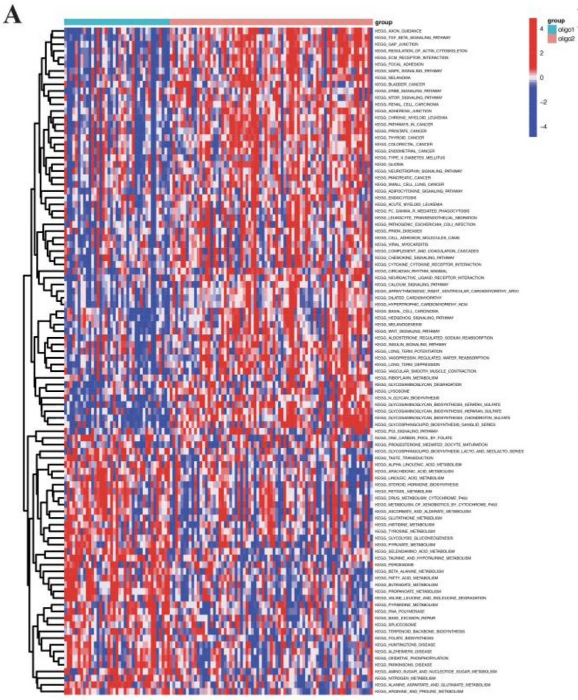

B

Dependent: Surv(time, state)		all	HR (univariable)	HR (multivariable)
gender	FEMALE	13 (14.1%)		
	MALE	79 (85.9%)	0.22 (0.09-0.50, p<.001)	0.25 (0.10-0.64, p=.004)
age	Mean ± SD	61.1 ± 7.9	0.98 (0.93-1.03, p=.456)	
Stage	Stage I	2 (2.2%)		
	Stage II	5 (5.4%)		
	Stage III	13 (14.1%)		
	Stage IV	72 (78.3%)		
T	T1	5 (5.4%)		
	T2	9 (9.8%)	0.68 (0.06-7.65, p=.758)	
	T3	25 (27.2%)	1.30 (0.16-10.50, p=.803)	
	T4	53 (57.6%)	1.43 (0.19-10.78, p=.729)	
N	N0	39 (42.4%)		
	N1	12 (13.0%)	2.28 (0.74-7.02, p=.151)	1.02 (0.29-3.57, p=.973)
	N2	39 (42.4%)	2.23 (0.96-5.19, p=.061)	1.71 (0.71-4.17, p=.234)
	N3	2 (2.2%)	55.41 (8.26-371.53, p<.001)	23.12 (2.57-207.96, p=.005)
riskScore	Mean ± SD	2.6 ± 4.1	1.25 (1.16-1.34, p<.001)	1.28 (1.17-1.41, p<.001)

n=92, events=33, Likelihood ratio test=50.18 on 5 df(p<.001)

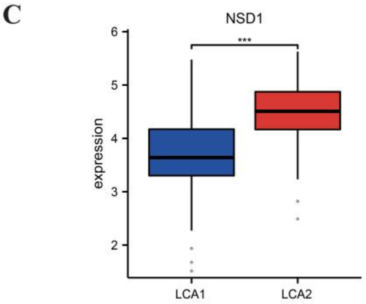

Fig. S4 (A) Heatmap showed the different results of KEGG enrichment analysis between the two subtypes in the TCGA cohorts (the pathways of P < 0.05 were shown in the heatmap, by Wilcon rank-sum test.) (B) Univariate and multivariate cox regression analysis exhibited the relationship between clinical features and overall survival in LCA patients. (C) The expression of *NSD1* appeared a significant reduce in *LCA1*.(*P < 0.05, **P < 0.01, ***P < 0.001, Wilcon rank-sum test.)

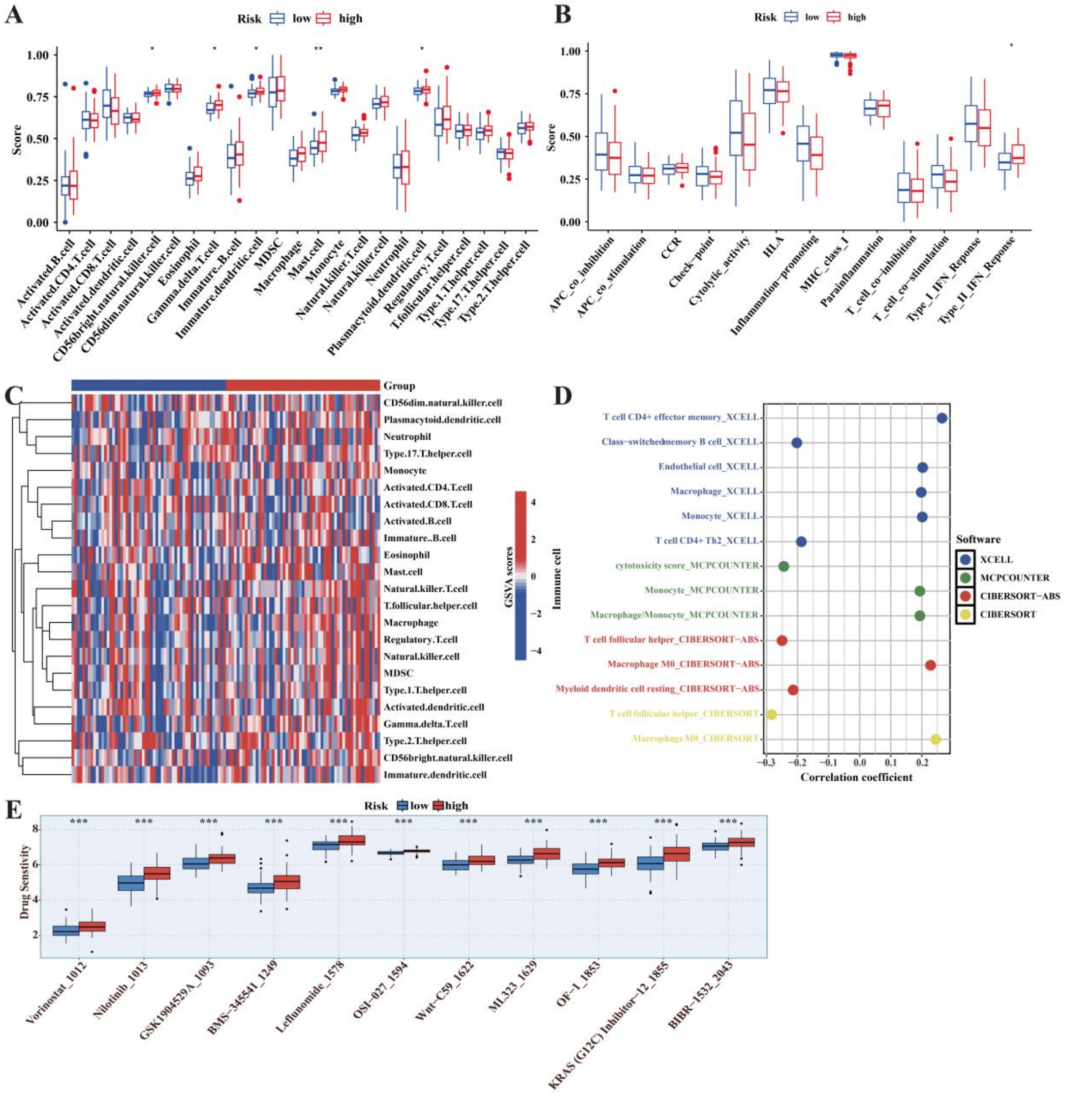

Fig. S5 Tumor immune infiltration and drug sensitivity differences between high-risk group and low-risk group. (A) Box plot comparing the 13 immune-related functions and 23 immune cells in “GSVA” package among two groups. (B) Heatmap shows differences of immune-related cells in two groups. (C) TIMER, XCELL, ABS, CIBERSORT-ABS, QUANTISEQ, MCPCOUNTER, EPIC and CIBERSORT analysis were used to perform correlation analysis between immune cells and RiskScore, results with p-value < 0.05 are shown. (D) OncoPredict was used to estimate the drug sensitivity differences between two groups, outcomes of p<0.001 were displayed. (*P < 0.05, **P < 0.01, ***P < 0.001, Wilcon rank-sum test.)

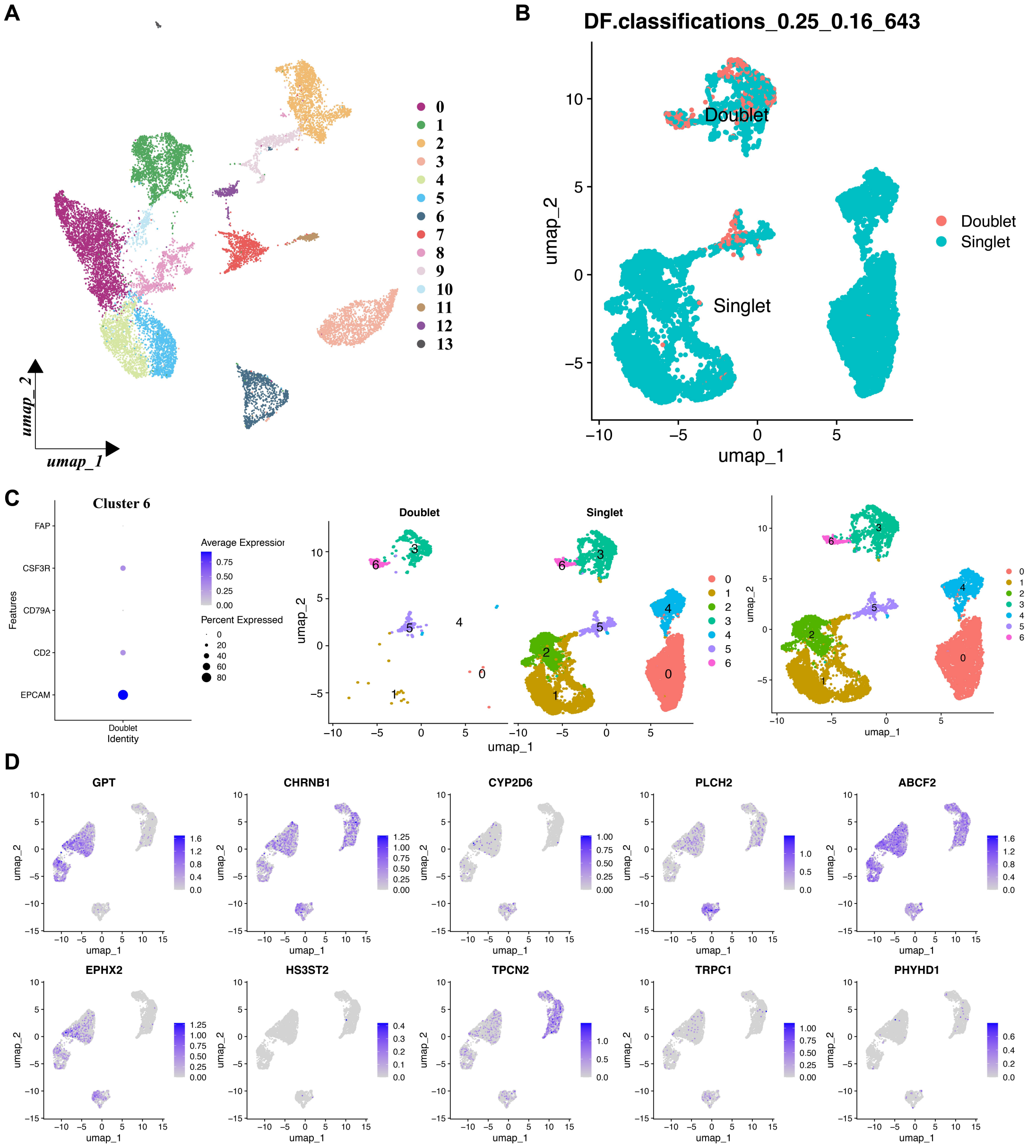

Fig. S6 (A) Umap plot showed the distribution of the 14 clusters of cells in laryngeal cancer tissues. (B) and (C) Doublets were identified and removed from the 6 cluster in epithelial-derived cells. (D) Feature plots presented the prognostic related genes' expressions for malignant epithelial cells.

Table S1 Clinical features differences of patients in the TCGA-LCA dataset between metabolic subtypes.

Covariates	Type	All	LCA1	LCA2	Pvalue
Gender	FEMALE	19(16.67%)	6(15.38%)	13(17.33%)	1
	MALE	95(83.33%)	33(84.62%)	62(82.67%)	
Age	<=65	74(64.91%)	28(71.79%)	46(61.33%)	0.3662
	>65	40(35.09%)	11(28.21%)	29(38.67%)	
Stage	Stage I	2(1.75%)	1(2.56%)	1(1.33%)	0.1727
	Stage II	9(7.89%)	2(5.13%)	7(9.33%)	
	Stage III	14(12.28%)	8(20.51%)	6(8%)	
	Stage IV	72(63.16%)	24(61.54%)	48(64%)	
	Stage IVC	1(0.88%)	1(2.56%)	0(0%)	
	unkown	16(14.04%)	3(7.69%)	13(17.33%)	
T	T1	7(6.14%)	3(7.69%)	4(5.33%)	0.7186
	T2	13(11.4%)	4(10.26%)	9(12%)	
	T3	26(22.81%)	11(28.21%)	15(20%)	
	T4	54(47.37%)	18(46.15%)	36(48%)	
	unkown	14(12.28%)	3(7.69%)	11(14.67%)	
N	N0	40(35.09%)	19(48.72%)	21(28%)	0.0891
	N1	12(10.53%)	5(12.82%)	7(9.33%)	
	N2	40(35.09%)	12(30.77%)	28(37.33%)	
	N3	2(1.75%)	0(0%)	2(2.67%)	
	unkown	20(17.54%)	3(7.69%)	17(22.67%)	
M	M0	39(34.21%)	14(35.9%)	25(33.33%)	0.3541
	M1	1(0.88%)	1(2.56%)	0(0%)	
	unkown	74(64.91%)	24(61.54%)	50(66.67%)	

Table S4 Clinical features differences of patients in TCGA-LCA dataset between the risk groups.

Covariates	Type	All	high	low	Pvalue
Gender	FEMALE	19(16.67%)	13(22.81%)	6(10.53%)	0.1316
	MALE	95(83.33%)	44(77.19%)	51(89.47%)	
Age	<=65	74(64.91%)	36(63.16%)	38(66.67%)	0.8444
	>65	40(35.09%)	21(36.84%)	19(33.33%)	
Stage	Stage I	2(1.75%)	0(0%)	2(3.51%)	0.2083
	Stage II	9(7.89%)	7(12.28%)	2(3.51%)	
	Stage III	14(12.28%)	5(8.77%)	9(15.79%)	
	Stage IV	72(63.16%)	36(63.16%)	36(63.16%)	
	Stage IVC	1(0.88%)	0(0%)	1(1.75%)	
	unkown	16(14.04%)	9(15.79%)	7(12.28%)	
T	T1	7(6.14%)	0(0%)	7(12.28%)	0.0654
	T2	13(11.4%)	8(14.04%)	5(8.77%)	
	T3	26(22.81%)	13(22.81%)	13(22.81%)	
	T4	54(47.37%)	27(47.37%)	27(47.37%)	
	unkown	14(12.28%)	9(15.79%)	5(8.77%)	
N	N0	40(35.09%)	16(28.07%)	24(42.11%)	0.0611
	N1	12(10.53%)	6(10.53%)	6(10.53%)	
	N2	40(35.09%)	18(31.58%)	22(38.6%)	
	N3	2(1.75%)	2(3.51%)	0(0%)	
	unkown	20(17.54%)	15(26.32%)	5(8.77%)	
M	M0	39(34.21%)	16(28.07%)	23(40.35%)	0.21
	M1	1(0.88%)	0(0%)	1(1.75%)	
	unkown	74(64.91%)	41(71.93%)	33(57.89%)	

RiskScore formular:

*RiskScore = h0(t) * exp (- 0.973* GPT expression + 0.680* HS3ST2 expression - 0.758* CHRNB1 expression - 1.021* CYP2D6 expression - 0.24* PLCH2 expression + 0.265* TPCN2 expression - 1.364* ABCF2 expression - 0.536* EPHX2 expression + 0.595* TRPC1 expression + 0.541* PHYHD1 expression) .*

(lnh0(t) = 8.89669276)
